# Bridging integrator 1 fragment accelerates tau aggregation and propagation by enhancing clathrin-mediated endocytosis in mice

**DOI:** 10.1371/journal.pbio.3002470

**Published:** 2024-01-11

**Authors:** Xingyu Zhang, Li Zou, Li Tang, Min Xiong, Xiao-Xin Yan, Lanxia Meng, Guiqin Chen, Jing Xiong, Shuke Nie, Zhaohui Zhang, Qiang Chen, Zhentao Zhang

**Affiliations:** 1 Department of Neurology, Renmin Hospital of Wuhan University, Wuhan, China; 2 Department of Neurology, Zhongnan Hospital of Wuhan University, Wuhan, China; 3 Department of Anatomy and Neurobiology, Central South University Xiangya School of Medicine, Changsha, China; 4 Frontier Science Center for Immunology and Metabolism, Medical Research Institute, Wuhan University, Wuhan, China; 5 TaiKang Center for Life and Medical Sciences, Wuhan University, Wuhan, China; Stony Brook University Medical Center: Stony Brook University Hospital, UNITED STATES

## Abstract

The bridging integrator 1 (BIN1) gene is an important risk locus for late-onset Alzheimer’s disease (AD). BIN1 protein has been reported to mediate tau pathology, but the underlying molecular mechanisms remain elusive. Here, we show that neuronal BIN1 is cleaved by the cysteine protease legumain at residues N277 and N288. The legumain-generated BIN1 (1–277) fragment is detected in brain tissues from AD patients and tau P301S transgenic mice. This fragment interacts with tau and accelerates its aggregation. Furthermore, the BIN1 (1–277) fragment promotes the propagation of tau aggregates by enhancing clathrin-mediated endocytosis (CME). Overexpression of the BIN1 (1–277) fragment in tau P301S mice facilitates the propagation of tau pathology, inducing cognitive deficits, while overexpression of mutant BIN1 that blocks its cleavage by legumain halts tau propagation. Furthermore, blocking the cleavage of endogenous BIN1 using the CRISPR/Cas9 gene-editing tool ameliorates tau pathology and behavioral deficits. Our results demonstrate that the legumain-mediated cleavage of BIN1 plays a key role in the progression of tau pathology. Inhibition of legumain-mediated BIN1 cleavage may be a promising therapeutic strategy for treating AD.

## Introduction

Alzheimer’s disease (AD) is the most common progressive neurodegenerative disorder. Pathologically, AD is characterized by the deposition of extracellular amyloid-β plaques and intraneuronal neurofibrillary tangles composed of aggregated tau. The extent of tau aggregation correlates better with the severity of neurodegeneration and cognitive impairment than amyloid deposits [1]. During the progression of AD, tau pathology usually starts in subcortical nuclei such as the locus coeruleus and then spreads to limbic regions, including the subiculum, hippocampal cornu ammonis (CA), and amygdala, before eventually progressing to the neocortex [2]. Converging evidence suggests that tau propagation may occur via a “prion-like” transmission mode [3–5], by which the aggregated tau induces the soluble tau monomers to form aggregates of the same conformation, initiating a self-amplifying cascade. Injection of human AD brain extracts containing tau aggregates into a mouse brain induces tau pathology, which spreads from the injection site to the brain regions anatomically connected to the injection site [3,4]. In vitro studies showed that extracellular tau aggregates can be taken up by cultured cells and “seed” the aggregation of soluble tau. Furthermore, pathological tau aggregates transfer between cells in a way similar to the propagation of prion protein [5–7]. However, the mechanisms underlying the propagation of tau pathology have yet to be elucidated.

Bridging integrator 1 (BIN1), a ubiquitously expressed protein, plays a pivotal role in multiple cellular processes, including endocytosis and trafficking, membrane recycling, cell cycle progression, apoptosis, and cytoskeleton regulation [8,9]. At least 10 isoforms of BIN1 are expressed in different tissues, with the longest isoform (isoform 1) being specifically expressed in the brain. Isoform 1 contains a CLAP domain that interacts with clathrin and adaptor protein 2 (AP2), thus playing a role in clathrin-mediated endocytosis (CME) [10,11]. Genome-wide association studies (GWAS) identified BIN1 as the second most significant genetic risk locus for sporadic AD [12,13]. The expression of brain-specific BIN1 is decreased in the brain tissue of patients with AD, while that of shorter BIN1 isoforms is increased. The expression of BIN1 correlates with neurofibrillary tangle pathology [14]. Furthermore, brain-specific BIN1 was found to alleviate tau pathology, whereas the down-regulation of BIN1 enhanced the propagation of tau pathology [9]. However, the exact role of BIN1 in the onset and progression of AD has not been elucidated.

Legumain is an endolysosomal cysteine protease that cleaves its substrates after asparagine (N) residues [15,16]. As has been shown before, legumain is activated in the human AD brain [17]. Legumain cleaves tau and amyloid precursor protein (APP), mediating the formation of neurofibrillary pathology and the generation of amyloid-β [17,18]. These studies suggest that legumain plays an important role in AD. Here, we further investigated whether legumain is involved in BIN1-mediated tau pathology. We show that legumain cleaves BIN1 at the N277 and N288 residues, with N277 being the major cleavage site in the brains of AD patients. The legumain-generated BIN1 (1–277) fragment promotes the uptake and propagation of tau aggregates by enhancing CME. Moreover, BIN1 (1–277) interacts with tau and accelerates the de novo assembly of tau fibrils. Overexpression of BIN1 (1–277) in tau P301S mice facilitates the propagation of tau pathology and induces behavioral defects. Blockage of legumain-mediated cleavage of BIN1 ameliorates the pathological and behavioral deficits in tau P301S mice.

## Results

### Legumain cleaves BIN1 in vitro

**Fig 1:**
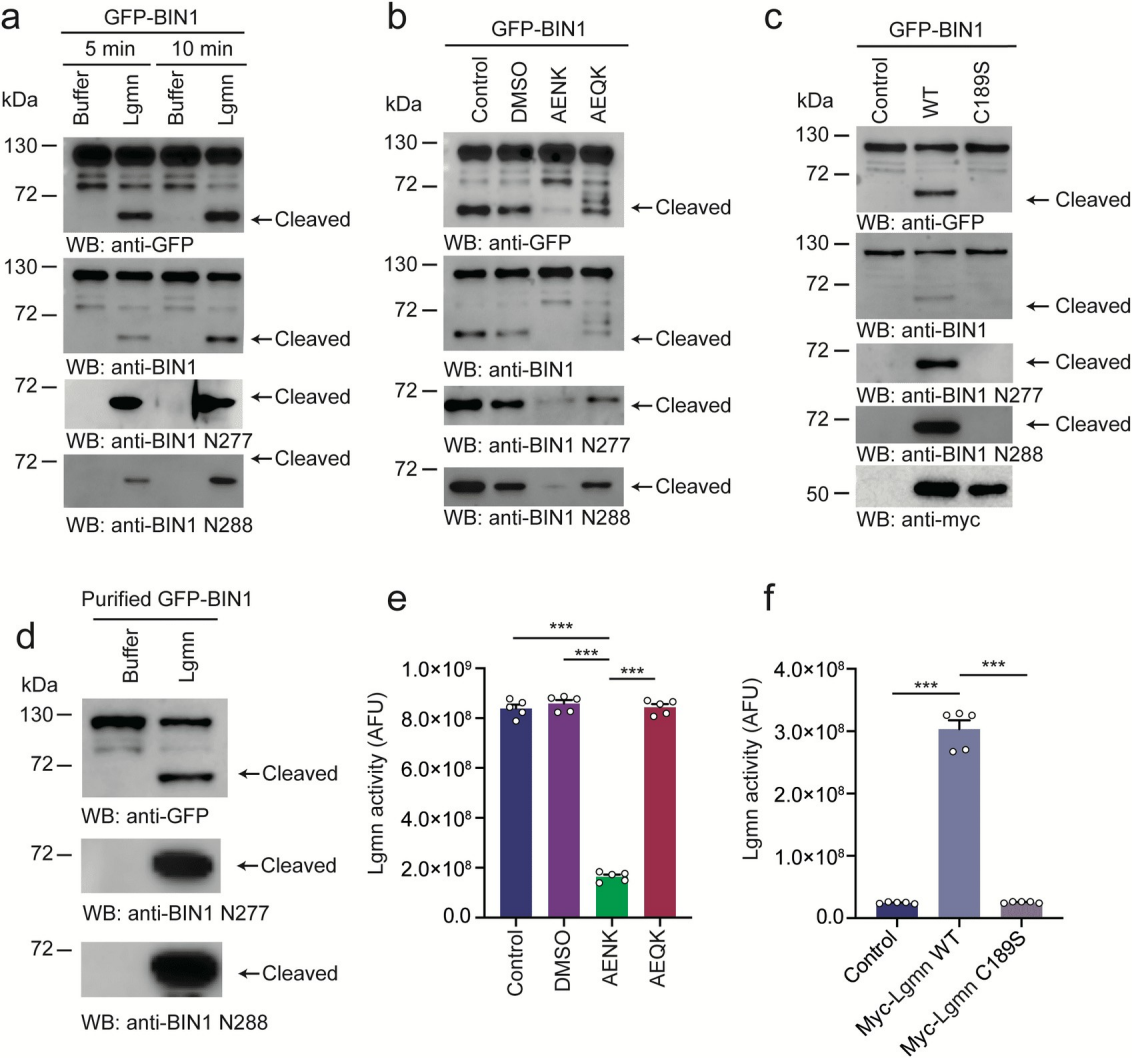
Legumain cleaves BIN1 in vitro. (**a**) GFP-BIN1 was incubated with legumain for 5 min and 10 min, respectively. WB shows the generation of the BIN1 fragment in a time-dependent manner. (**b**) The legumin inhibitor AENK blocks the fragmentation of GFP-BIN1. (**c**) Cleavage of GFP-BIN1 by WT and C189S mutant legumain. (**d**) WB shows the cleavage of purified BIN1 by legumain. (**e**) Legumain activity was inhibited by AENK but not AEQK. Data represent mean ± SEM of 5 independent experiments. ***P < 0.001. (**f**) The C189S mutation diminishes legumain activity. Data represent mean ± SEM of 5 independent experiments. ***P < 0.001. Source data can be found in S1 Data and S1 Raw Images. AFU, arbitrary fluorescence unit; BIN1, bridging integrator 1; DMSO, dimethyl sulfoxide; GFP, green fluorescent protein; MW, molecular weight; WB, western blot; WT, wild-type.

To explore whether legumain cleaves BIN1 in vitro, we incubated GFP-BIN1 with active legumain. A truncated BIN1 band (approximately 60 kDa) was detected in a time-dependent manner (Figs 1A and S1A). Furthermore, GFP-BIN1 was fragmented when incubated in mouse brain lysates at pH 6.0 (S1A Fig). The appearance of this truncated band was inhibited by the legumain inhibitor AENK but not the inactive analog AEQK (Fig 1B). Furthermore, wild-type legumain strongly triggered BIN1 fragmentation, whereas legumain with a C189S protease-inactive mutant was unable to provoke BIN1 cleavage (Fig 1C). These results indicate that inactivation of legumain blocks BIN1 fragmentation in vitro. Moreover, purified BIN1 was also cleaved by purified active legumain, indicating that legumain directly cleaves BIN1 (Fig 1D). The legumain activity assay confirmed that the peptide inhibitor AENK or the C189S mutant abolished the enzymatic activity of legumain (Fig 1E and 1F). Together, these results indicate that BIN1 is a substrate of legumain.

### Legumain cleaves BIN1 at the N277 and N288 residues

**Fig 2:**
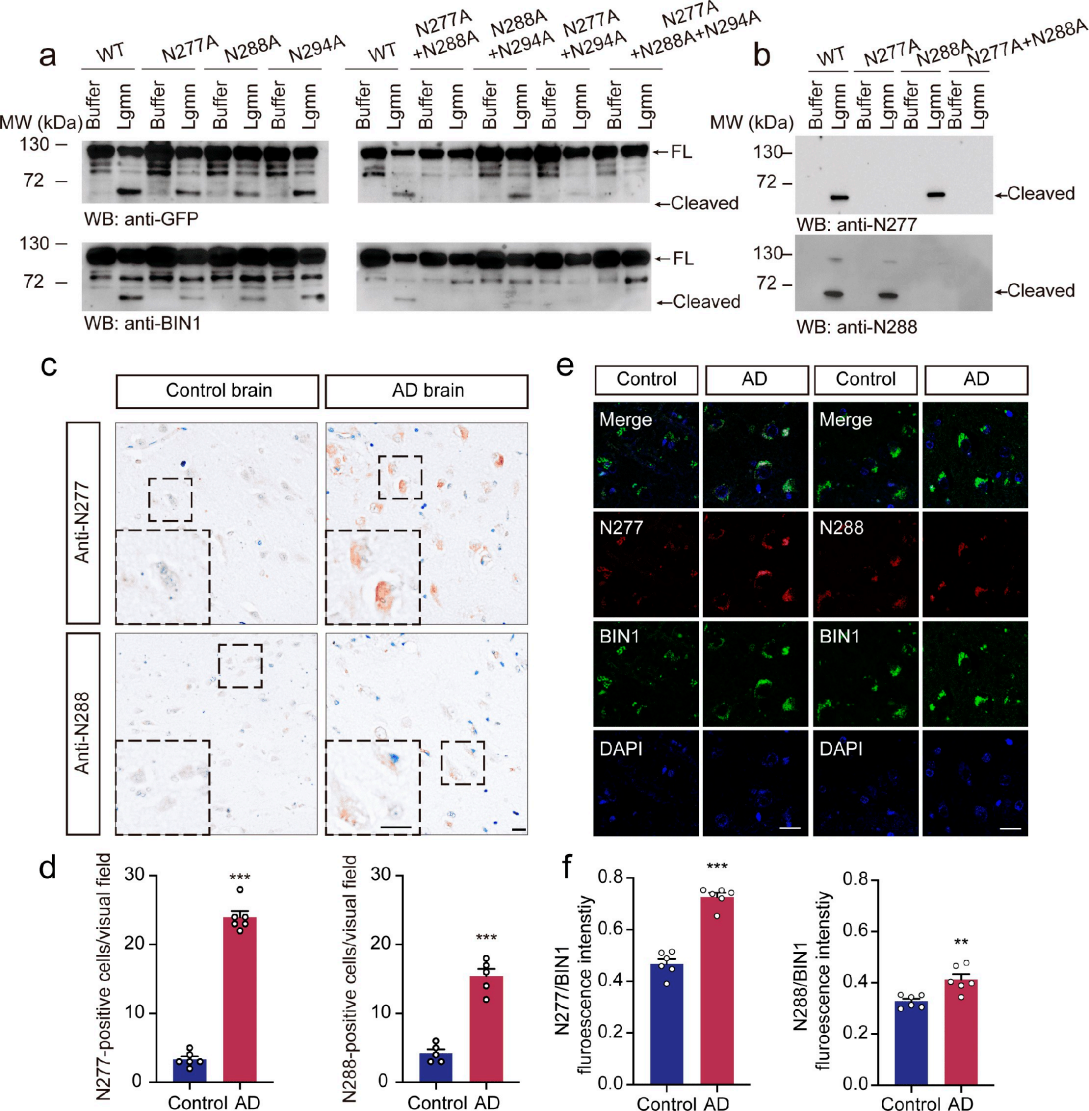
Legumain cleaves BIN1 at N277 in the AD brain. (**a**) Cleavage of mutant BIN1 by legumain. Fragmentation was blocked by the N277A/N288A mutation. (**b**) Cleavage of mutant BIN1 by legumain detected by N277 and N288 fragment-specific antibodies. (**c**, **d**) IHC of BIN1 N277 and N288 fragments in brain sections from patients with AD and control subjects (mean ± SEM; Student *t* test, *n* = 6 patients per group; ***P < 0.001). Scale bar, 20 μm. (**e**) IF of BIN1, N277, and N288 fragments in brain sections from AD patients and control subjects. Scale bar, 20 μm. (**f**) Quantification of N277/BIN1 and N288/BIN1 fluorescence intensity relative to the control group in (**e**) (mean ± SEM; Student *t* test, *n* = 6 patients per group; **P < 0.01, ***P < 0.001). Source data can be found in S1 Data and S1 Raw Images. AD, Alzheimer’s disease; BIN1, bridging integrator 1; FL, full-length; GFP, green fluorescent protein; IF, immunofluorescence; IHC, immunohistochemistry; MW, molecular weight; WB, western blot; WT, wild-type.

Legumain specifically cleaves its substrates after N residues. Thus, we introduced a series of point mutations, including BIN1 N277A, N288A, and N299A, to identify the BIN1 cleavage site. Mutation of either N277A or N288A partially blocked the appearance of the band at 60 kDa, while the double mutant (N277A/N288A) completely blocked the cleavage. The N294A mutant construct was efficiently cleaved by legumain (Fig 2A). These results suggest that BIN1 is cleaved at N277 and N288. To distinguish the 2 BIN1 fragments generated by legumain, we developed 2 antibodies that specifically recognize the BIN1 (1–277) and BIN1 (1–288) fragments, respectively, but do not recognize the full-length protein or other bands (Figs 1A–2D, 2B and S1B). Preincubation with antigen peptides abolished the immunoreactivity. Furthermore, staining was detected in brain sections from wild-type mice but not in those from legumain knockout mice (S1C Fig). The omission of the primary or secondary antibodies diminished the staining (S1D Fig). These results support the specificity of the anti-BIN1 N277 and anti-BIN1 N288 antibodies. Both the BIN1 (1–277) and BIN1 (1–288) fragments were detected in human AD brain sections, but the signals were barely detected in the age-matched control brain sections. Furthermore, the BIN1 (1–277) immunoreactive fragment was more abundant than BIN1 (1–288) in the brains of AD patients (Fig 2C and 2D). The results of immunofluorescence staining also demonstrated that the levels of the BIN1 (1–277) fragment relative to full-length BIN1 were increased in the frontal cortex of human AD brains compared with age-matched control brain sections, while the signal of BIN1 (1–288) remained low (Fig 2E and 2F). In addition, the BIN1 N277 fragment was detected in neurons, microglia, astrocytes, and oligodendrocytes (S1E Fig). Western blot analysis confirmed the presence of BIN1 (1–277) fragments in the brain lysates from AD patients (S2A and S2B Fig). To determine the relationship between the levels of the BIN1 (1–277) fragment and tau pathology, we detected their levels in brain tissue of different Braak stages. Both tau pathology and the level of the BIN1 (1–277) fragment increased during the progression of AD (S2C–S2E Fig). Furthermore, the levels of BIN1 (1–277) correlated with the levels of p-Tau (S2F Fig). In addition, we found that the levels of the BIN1 (1–277) fragment increased in tau P301S mice in an age-dependent manner, while the levels of BIN1 (1–288) remained low (S3A–S3F Fig). Hence, legumain cleaves BIN1 at N277 and N288 in the brain, with the former being the major cleavage site. Thus, we focused on BIN1 (1–277) in our study.

### The BIN1 (1–277) fragment accelerates the uptake of tau aggregates

**Fig 3:**
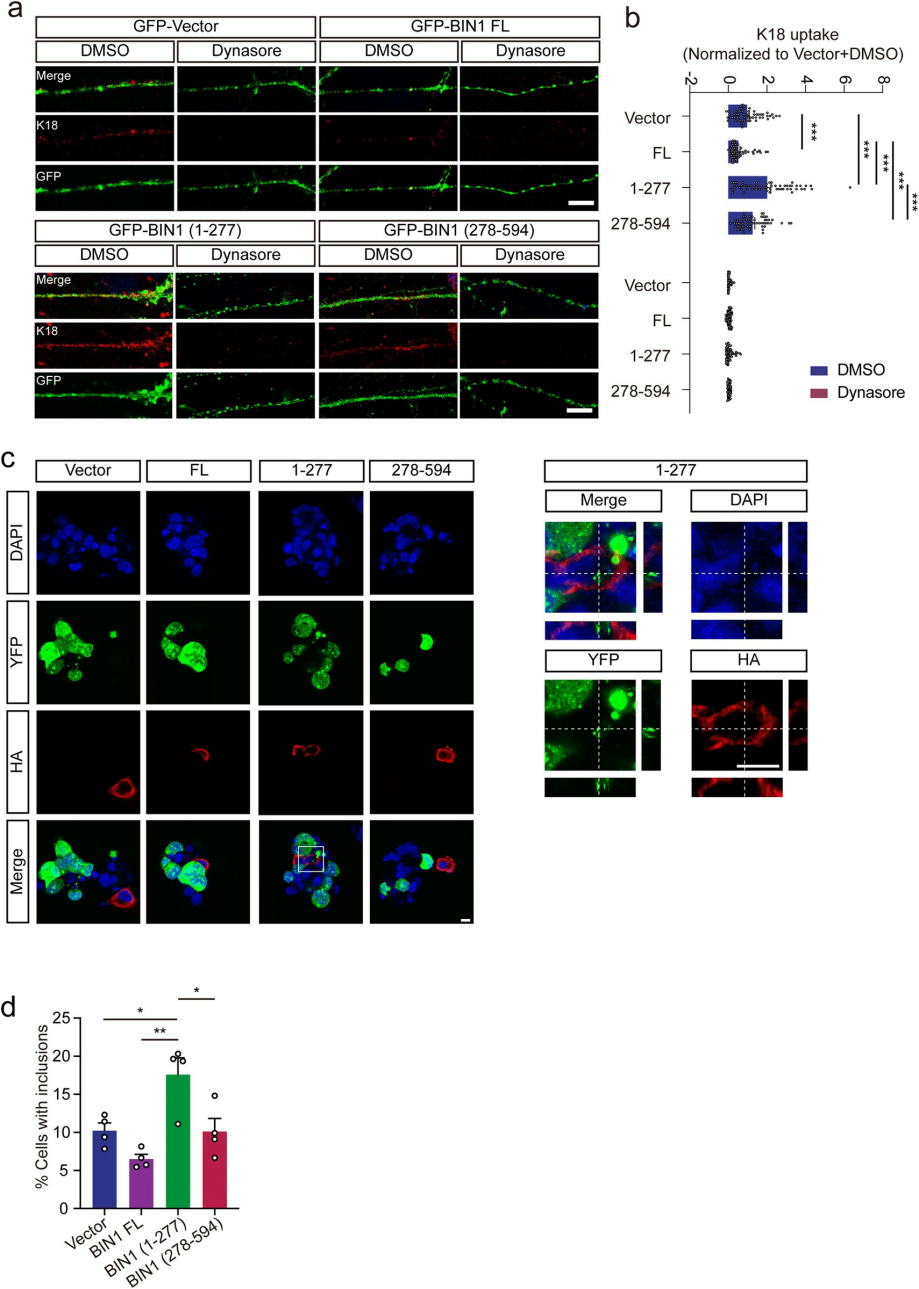
BIN1 (1–277) promotes the propagation of tau pathology. (**a**, **b**) Representative immunofluorescence images showing the uptake of K18 fibrils by neurons expressing EGFP, BIN1, BIN1 (1–277), and BIN1 (278–594). Neurons treated with dynasore were used as negative control. Scale bar, 5 μm. Quantification of K18 fibril uptake (**b**). The uptake of K18 fibrils was calculated as the mean fluorescence density of 60 cells under each condition. The fluorescence density was normalized to the Vector+DMSO group (mean ± SEM *n* = 60 cells per group; ***P < 0.001). (**c**) Transmission of tau aggregates from the donor cells to COS-7 cells transfected with HA-vector, BIN1, BIN1 (1–277), and BIN1 (278–594). The right panel shows the three-dimensional image of cells transfected with HA-BIN1 (1–277). Scale bar, 20 μm. (**d**) Quantification of the percentage of cells containing aggregates in cells expressing HA-vector, BIN1, BIN1 (1–277), or BIN1 (278–594). Data represent mean ± SEM of 4 independent experiments. *P < 0.05, **P < 0.01. Source data can be found in S1 Data. BIN1, bridging integrator 1; DMSO, dimethyl sulfoxide; EGFP, enhanced green fluorescent protein; FL, full-length; HA, human influenza hemagglutinin; YFP, yellow fluorescent protein.

Since BIN1 is an endocytosis-related protein, we further tested whether the legumain-generated BIN1 fragments influence the uptake of pathological tau in primary cultured neurons. The purity of the neurons was confirmed by immunostaining with the neuronal marker MAP2, astrocyte marker GFAP, and microglial marker Iba1 (S4A Fig). The neurons were infected with adeno-associated virus (AAV) encoding GFP-tagged BIN1, BIN1 (1–277), and BIN1 (278–594), respectively. GFP signals were detected only in neurons that stained positive for MAP2 (S4A Fig). The cell viability of neurons expressing full-length BIN1 or its fragments was similar (S4B Fig). The neurons were then exposed to tau RD fibrils. The uptake of tau RD fibrils was significantly enhanced in the presence of the BIN1 (1–277) fragment (Fig 3A and 3B). To investigate whether BIN1 fragments alter the degradation rate of tau, neurons were exposed to K18 fibrils for 30 min and washed with PBS to eliminate free fibrils. The K18 signals in neurons were observed at different time points after washing. There was no difference in degradation rates among the 4 groups (S5A and S5B Fig), indicating that increased signals in BIN1 (1–277) were not due to impairment of the degradation system.

We further detected the cell-to-cell propagation of tau aggregates in a coculture system. COS-7 cells were transfected with HA-BIN1, HA-BIN1 (1–277), or HA-BIN1 (278–594) and cocultured with HEK293 cells that consistently contain tau inclusions [19]. The cells expressing BIN1 (1–277) exhibited more inclusions translated from the donor cells than the other groups, which was confirmed by the three-dimensional image rendered from the Z-stack (Fig 3C and 3D). Overall, these results demonstrate that BIN1 (1–277) promotes the uptake of tau fibrils and enhances the cell-to-cell transmission of tau pathology.

### The BIN1 (1–277) fragment promotes CME

**Fig 4:**
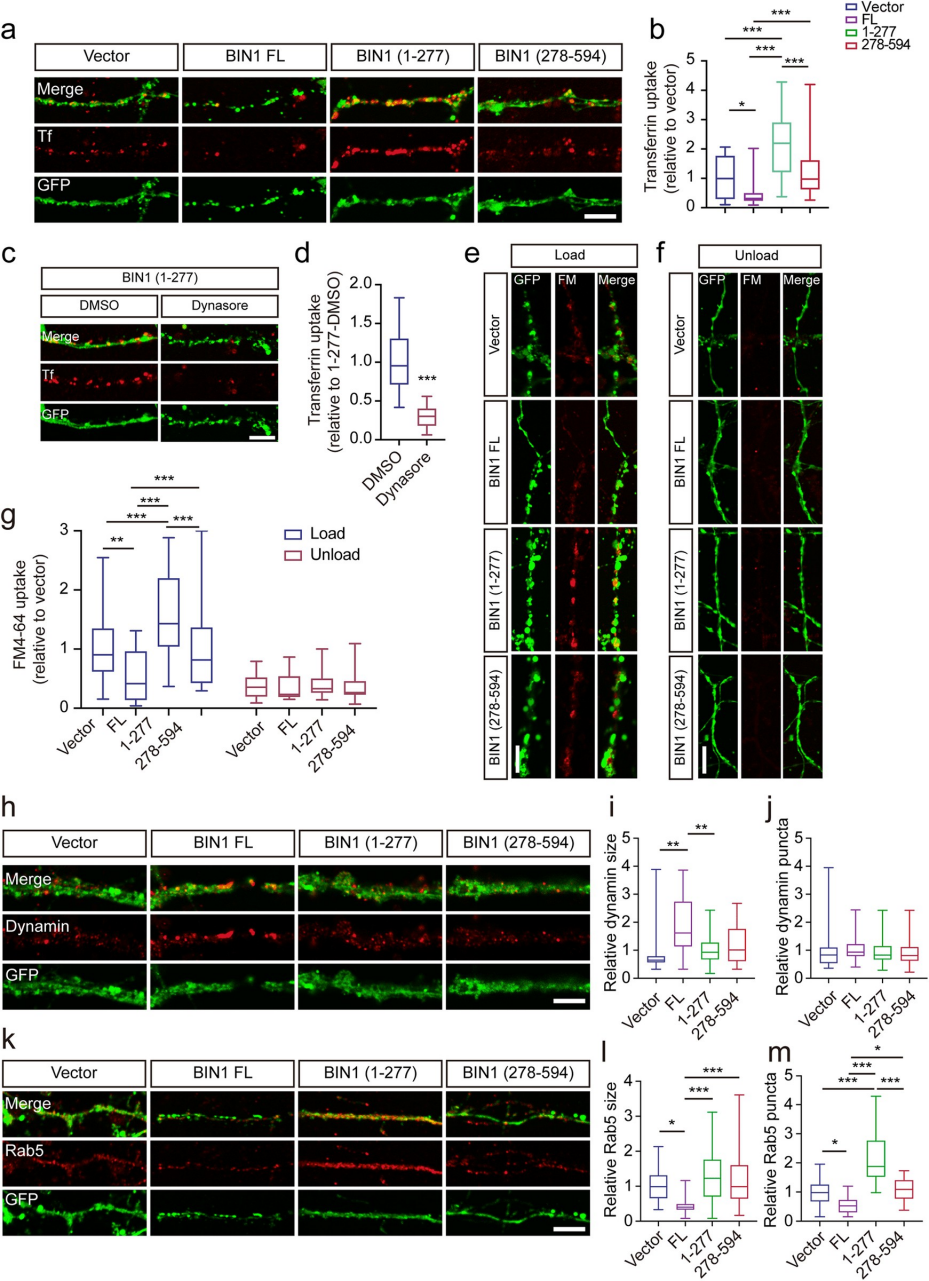
BIN1 (1–277) enhances CME. (**a**) Transferrin uptake assay of neurons expressing EGFP, BIN1, BIN1 (1–277), and BIN1 (278–594). Scale bar, 5 μm. (**b**) Quantification of transferrin uptake in (**a**). The uptake of transferrin was calculated as the mean fluorescence density of 30 cells under each condition. The fluorescence density was normalized to the GFP-Vector group (mean ± SEM *n* = 30 cells; *P < 0.05, ***P < 0.001). (**c**, **d**) Transferrin uptake assay of neurons expressing BIN1 (1–277) in the presence or absence of dynamin inhibitor dynasore. Scale bar, 5 μm. (**d**) Quantification of transferrin uptake in (**c**). Mean ± SEM *n* = 30 cells; ***P < 0.001. (**e**) FM 4–64 uptake assay in neurons expressing EGFP-vector, BIN1, BIN1 (1–277), and BIN1 (278–594). Scale bar, 5 μm. (**f**) FM 4–64 dye after KCl-induced loss of fluorescence in neurons expressing EGFP-vector, BIN1, BIN1 (1–277), and BIN1 (278–594). Scale bar, 5 μm. (**g**) Quantification of FM 4–64 uptake. FM 4–64 labeling was calculated as the integral fluorescence intensity of 30 boutons under each condition. The fluorescence density was normalized to the control group (mean ± SEM *n* = 30 cells; *P < 0.05, **P < 0.01. ***P < 0.001). (**h**) Immunofluorescence showing the distribution of dynamin in neurons expressing EGFP, EGFP-BIN1, EGFP-BIN1 (1–277), and EGFP-BIN1 (278–594). Scale bar, 5 μm. (**i**, **j**) Quantification of the number and size of the dynamin puncta (mean ± SEM *n* = 30 cells; *P < 0.05). (**k**) Immunofluorescence showing the expression of Rab5 in neurons expressing EGFP, EGFP-BIN1, EGFP-BIN1 (1–277), and EGFP-BIN1 (278–594). Scale bar, 5 μm. (**l**, **m**) Quantification of the number and size of the Rab5 puncta (mean ± SEM *n* = 30; *P < 0.05, ***P < 0.001). Source data can be found in S1 Data. BIN1, bridging integrator 1; CME, clathrin-mediated endocytosis; DMSO, dimethyl sulfoxide; EGFP, enhanced green fluorescent protein; FL, full-length; FM, FM 4-64 dye; GFP, green fluorescent protein.

BIN1 interacts with clathrin, AP2, and dynamin and regulates CME, a process involved in the uptake of tau fibrils in AD [20,21]. We investigated whether the legumain-mediated fragmentation of BIN1 regulates the CME process using transferrin uptake assay in primary neurons (Fig 4A). Interestingly, overexpression of EGFP-BIN1 (1–277) enhanced transferrin uptake (Fig 4A and 4B). The uptake of transferrin was blocked by the endocytosis inhibitor dynasore in neurons expressing BIN (1–277) (Fig 4C and 4D). The uptake of tau fibrils was also inhibited by dynasore (Fig 3A). We then tested the uptake of FM 4–64 dye (FM) by neurons and found that BIN1 (1–277) dramatically enhanced the uptake of the FM 4–64 dye, while KCl-induced loss of fluorescence was not influenced by BIN1 fragments (Fig 4E–4G). These results indicate that the BIN1 (1–277) fragment promotes CME and the uptake of tau fibrils.

Considering that BIN1 interacts with dynamin and sequesters dynamin to inhibit CME [22], we assessed whether legumain-fragmented BIN (1–277) plays a different role in this process. Consistent with the previous report [22], full-length BIN1 increased the dynamin puncta size, but BIN1 (1–277) did not affect the size of dynamin puncta (Fig 4H–4J). In addition, the Glutathione S-Transferase (GST) pull-down assay demonstrated that BIN1 (1–277) failed to interact with dynamin as full-length BIN1 did (S5C Fig). These results indicate that BIN1 (1–277) loses the physiological function of full-length BIN1 to sequester dynamin. We further tested the expression of the early endosome marker Rab5. Overexpression of full-length BIN1 resulted in a decreased number and smaller size of Rab5-positive endosomes, while BIN1 (1–277) increased the number and size of Rab5 puncta relative to full-length BIN1 (Fig 4K–4M). These results imply that the BIN1 (1–277) fragment abolishes the inhibitory effect of full-length BIN1 on endocytosis.

### BIN1 (1–277) interacts with tau and promotes tau aggregation

**Fig 5:**
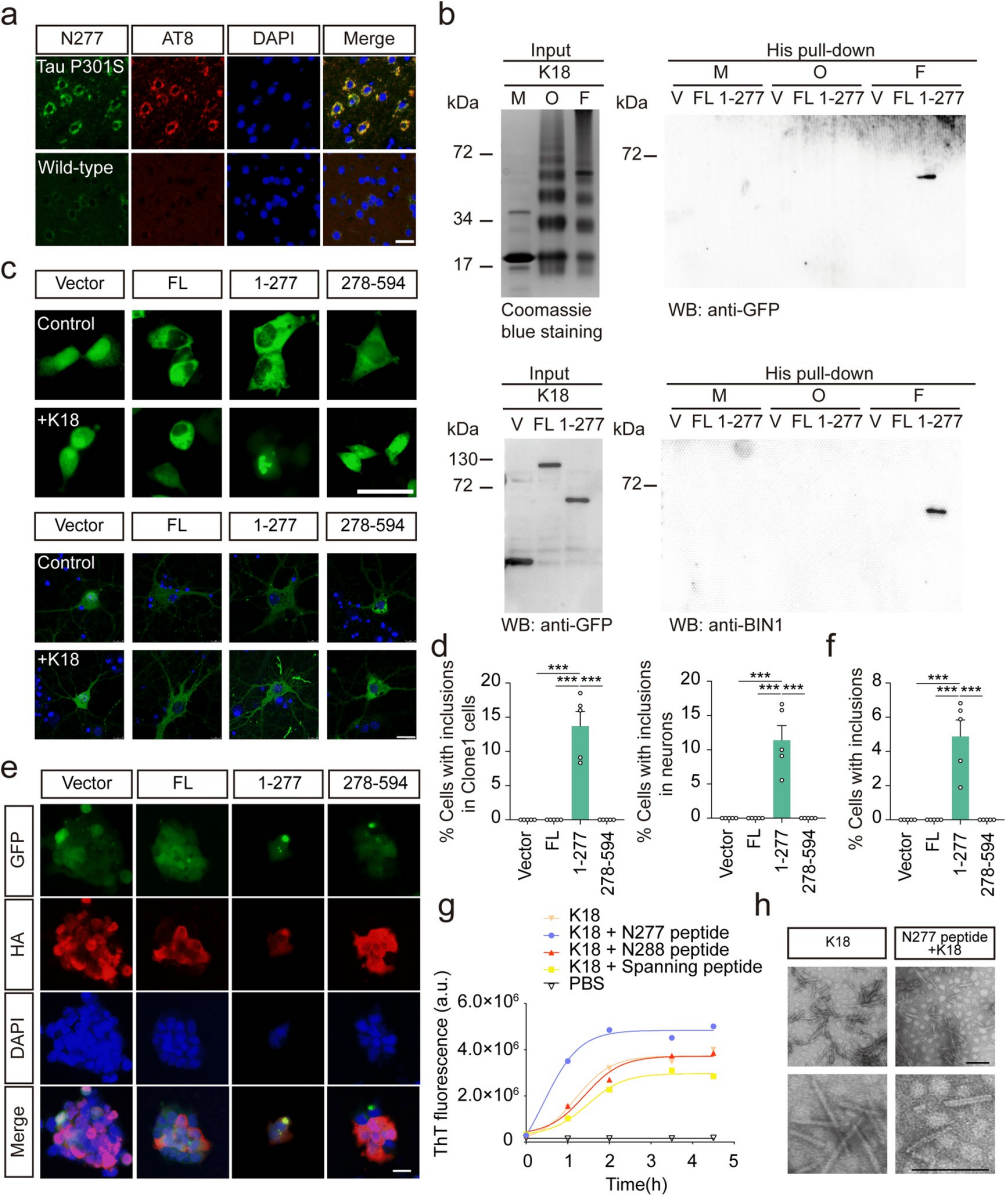
BIN1 (1–277) binds tau and facilitates its assembly. (**a**) Colocalization of AT8 and BIN1 (1–277) in the cortex and hippocampus of tau P301S mice. Scale bar, 20 μm. (**b**) His pull-down assay showing the interaction between BIN1 (1–277) and K18 fibrils. (**c**) BIN1 (1–277) enhances the seeding activity of K18 fibrils in clone 1 cells (upper panel) and primary neurons from tau P301S mice (lower panel). Scale bar, 20 μm. (**d**) The percentage of cells with inclusions in clone 1 cells (left panel) and primary neurons from tau P301S mice (right panel) (mean ± SEM *n* = 5 independent experiments. ***P < 0.001). (**e**, **f**) The Clone1 cells expressing HA-Vector, HA-BIN1 FL, HA-BIN1 (1–277), or HA-BIN1 (278–594) were transduced with K18 fibrils (1 μM) for 24 h. Scale bar, 20 μm. The bar graph shows the percentage of cells with inclusions (mean ± SEM *n* = 5 independent experiments. ***P < 0.001). (**g**) ThS assay showing the assembly kinetics of K18 in the presence of the BIN1 N277 peptide (265–277), BIN1 N288 peptide (265–287), and BIN1 spanning peptide (282–294), respectively. (**h**) Electron micrographs of the fibrils formed by incubating K18 for 12 h in the presence or absence of BIN1 N277 peptide. Scale bar, 120 nm. Source data can be found in S1 Data and S1 Raw Images. a.u., arbitrary unit; BIN1, bridging integrator 1; F, fibrils; FL, full-length; GFP, green fluorescent protein; HA, human influenza hemagglutinin; M, monomers; O, oligomers; WB, western blot.

To determine whether BIN1(1–277) directly interacts with tau and regulates its aggregation, brain sections from tau P301S transgenic mice and age-matched control mice were stained with phospho-tau (p-tau) and BIN1 (1–277) antibodies, revealing that BIN1 (1–277) colocalized with p-tau (Fig 5A). Furthermore, a pull-down assay found that BIN1 (1–277) interacts with fibrils (F) formed by the tau repeat domain (RD, K18) but not K18 monomers (M) or oligomers (O). We further overexpressed GFP-Vector, GFP-BIN1 full-length (FL), GFP-BIN1 (1–277), or GFP-BIN1 (278–594) in HEK293 cells or primary neurons derived from the tau P301S mouse brain. We found that GFP-BIN1 (1–277) aggregated into inclusions after transduction of the K18 fibrils (Fig 5C and 5D). These results suggest that K18 fibrils may initiate the assembly of BIN1 (1–277). Furthermore, we explored the effect of BIN1 (1–277) on the aggregation of tau using HEK293 cells stably expressing the GPF-tagged tau RD as reporter cells, as intracellular inclusions are formed when reporter cells are transduced with tau K18 fibrils [19]. We found that aggregates formed after expressing HA-BIN1 (1–277), and the aggregates of tau RD colocalized with HA-BIN1 (1–277), indicating that BIN1 (1–277) and tau may assemble together (Fig 5E and 5F).

To confirm the effect of BIN1 (1–277) on the aggregation of tau in vitro, we recorded the kinetics of amyloid assembly by tau-K18 in the presence or absence of the BIN1 N277 peptide (265–277 aa). The thioflavin S (ThS) fluorescence assay found that the BIN1 N277 peptide dramatically promoted the aggregation of tau-K18, with shorter lag times, steeper elongation phases, and higher final signals than tau-K18 alone, while the BIN1 spanning peptide (282–294 aa) and BIN1 N288 peptide (265–288 aa) showed no effect on the kinetics of K18 aggregation (Fig 5G). Under electron microscopy, the mixed fibrils consisting of K18 and N277 peptide were longer than the K18 fibrils and were highly ordered with a paired arrangement (Fig 5H), indicating that the BIN1 N277 peptide accelerates tau assembly and generates ordered paired helical structures. Co-sedimentation analysis detected the presence of both tau and BIN1 N277 peptide in the pellet fractions (S6A and S6B Fig). We further tested the seeding activity of K18 fibrils formed in the presence or absence of the BIN1 N277 peptide. When transduced into reporter cells, the BIN1 (1–277)-K18 fibrils induced more inclusions than the K18 fibrils formed in the absence of the BIN1 N277 peptide (S6C Fig). Overall, these results indicate that the BIN1 (1–277) fragment interacts with tau and accelerates its aggregation.

### BIN1 (1–277) promotes the propagation of tau pathology in vivo

**Fig 6:**
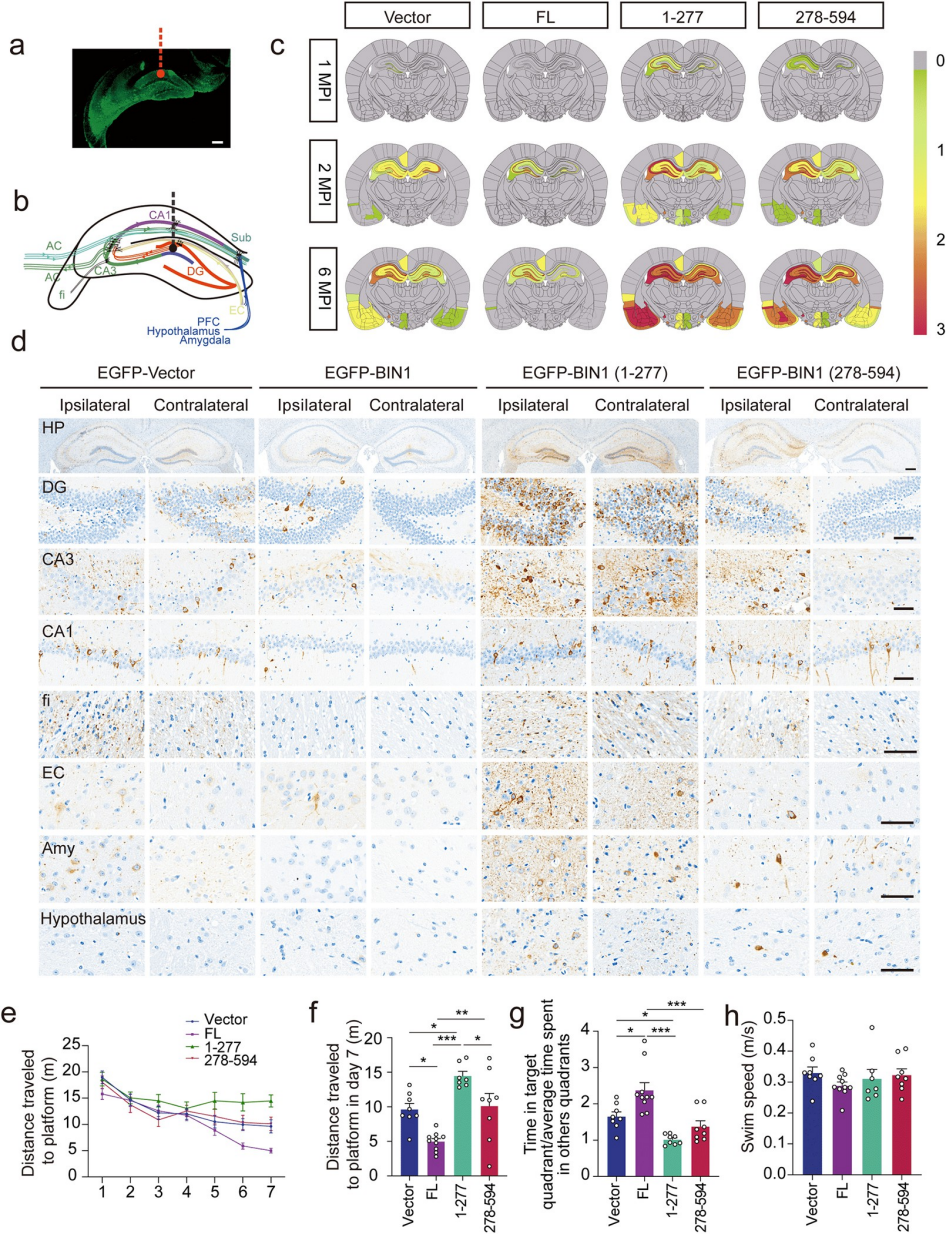
BIN1 (1–277) promotes tau pathology in tau P301S mice. (**a**) The expression of EGFP in tau P301S mice injected with AAV-EGFP. Scale bar, 200 μm. (**b**) Schematic of the hippocampal network. (**c**) Semiquantitative heatmap of tau pathology in tau P301S mice injected with AAVs encoding BIN1 FL, (1–277), and (278–594), respectively. (**d**) AT8 immunostaining of the DG, CA3, CA1, fi, EC, amygdala, and hypothalamus in tau P301S mice 6 months after injection. Scale bar of the whole HP, 280 μm; scale bar of other regions, 50 μm. (**e**, **f**) Morris water maze analysis of the distance traveled to the platform (**e**) and the distance traveled to the platform on day 7 (**f**) (mean ± SEM; *n* = 7–10 mice per group, *P < 0.05, **P < 0.01. ***P < 0.001). (**g**) Probe trial of the Morris water maze test analyzed as time spent in the target quadrant versus the average of time spent in other quadrants (mean ± SEM; *n* = 7–10; *P < 0.05, ***P < 0.001, one-way ANOVA). (**h**) Swimming speed of mice injected with AAVs-EGFP-Vector, BIN1-FL, BIN1 (1–277), and BIN1 (278–594) (mean ± SEM; *n* = 7–10 mice per group; one-way ANOVA). Source data can be found in S1 Data. AC, association commissural; AAV, adeno-associated virus; BIN1, bridging integrator 1; DG, dentate gyrus; EC, entorhinal cortex; EGFP, enhanced green fluorescent protein; FL, full-length; HP, hippocampus; MPI, month after injection; PFC, prefrontal cortex; Sub, subiculum.

Injection of tau fibrils into the brains of tau P301S transgenic mice induces the spreading of tau pathology to the structurally connected brain areas [4]. To explore whether BIN1 (1–277) regulates the propagation of tau pathology in vivo, we injected tau fibrils together with AAV particles encoding EGFP, EGFP-BIN1, EGFP-BIN1 (1–277), and EGFP-BIN1 (278–594) into the dentate gyrus (DG) of 2-month-old tau P301S mice. The expression of BIN1 and its fragments was confirmed by western blotting and immunofluorescence (Figs 6A, S7A and S7B). We examined the spreading of tau pathology in brain regions connected to the DG, including the hippocampal CA3, CA1, fimbria (fi), entorhinal cortex (EC), amygdala, and hypothalamus (Figs 6B and S8). One month after injection, tau pathology was detected in the ipsilateral DG area in mice expressing BIN1 (1–277) and BIN1 (278–594), but not in mice expressing EGFP or full-length BIN1. The mice expressing BIN (1–277) showed the most severe tau pathology (Figs 6C and S9A). Two months after injection, tau pathology was observed in the DG and CA3 areas in all mice except those overexpressing full-length BIN1, suggesting that full-length BIN1 halts, but the BIN1 (1–277) fragment promotes the propagation of tau pathology. Again, the mice expressing BIN1 (1–277) showed the most severe tau pathology among all the mice analyzed (Figs 6C and S9B). Tau pathology readily spread throughout the DG, CA3, and CA1 areas on both the ipsilateral and contralateral sides in the mice expressing BIN1 (1–277) (Figs 6C and S9B). At 6 months after injection, the CA1 area on both the ipsilateral and contralateral sides of mice injected with BIN1 (1–277) exhibited significant tau hyperphosphorylation. Furthermore, the contralateral side of mice expressing full-length BIN1 exhibited minimal tau pathology (Fig 6C and 6D). The burden of tau pathology was significantly increased in the fi, EC, amygdala, and hypothalamus in the presence of BIN1 (1–277) (Fig 6C and 6D). These results suggest that BIN1 (1–277) promotes the propagation of tau pathology. Tau pathology was also observed in the mouse brain injected with BIN1 (278–594), suggesting that BIN1 (278–594) also slightly promotes the spreading of tau pathology.

The Morris water maze test found that the learning abilities of mice expressing BIN1 (1–277) were impaired compared with those of mice in other groups, as these mice traveled longer distances to find the platform during the training phase (Fig 6E and 6F) and spent less time in the target quadrant during the probe trial (Fig 6G). The swim speeds of all mice were comparable (Fig 6H), suggesting that BIN1 and its fragments do not affect motor function. The densities of synapses and dendritic spines were reduced in the hippocampus of mice injected with BIN1 (1–277) (S10A–S10D Fig). These results indicate that the BIN1 fragments facilitate the spreading of tau pathology and cause cognitive impairments.

### Overexpression of uncleavable BIN1 ameliorates tau pathology in vivo

**Fig 7:**
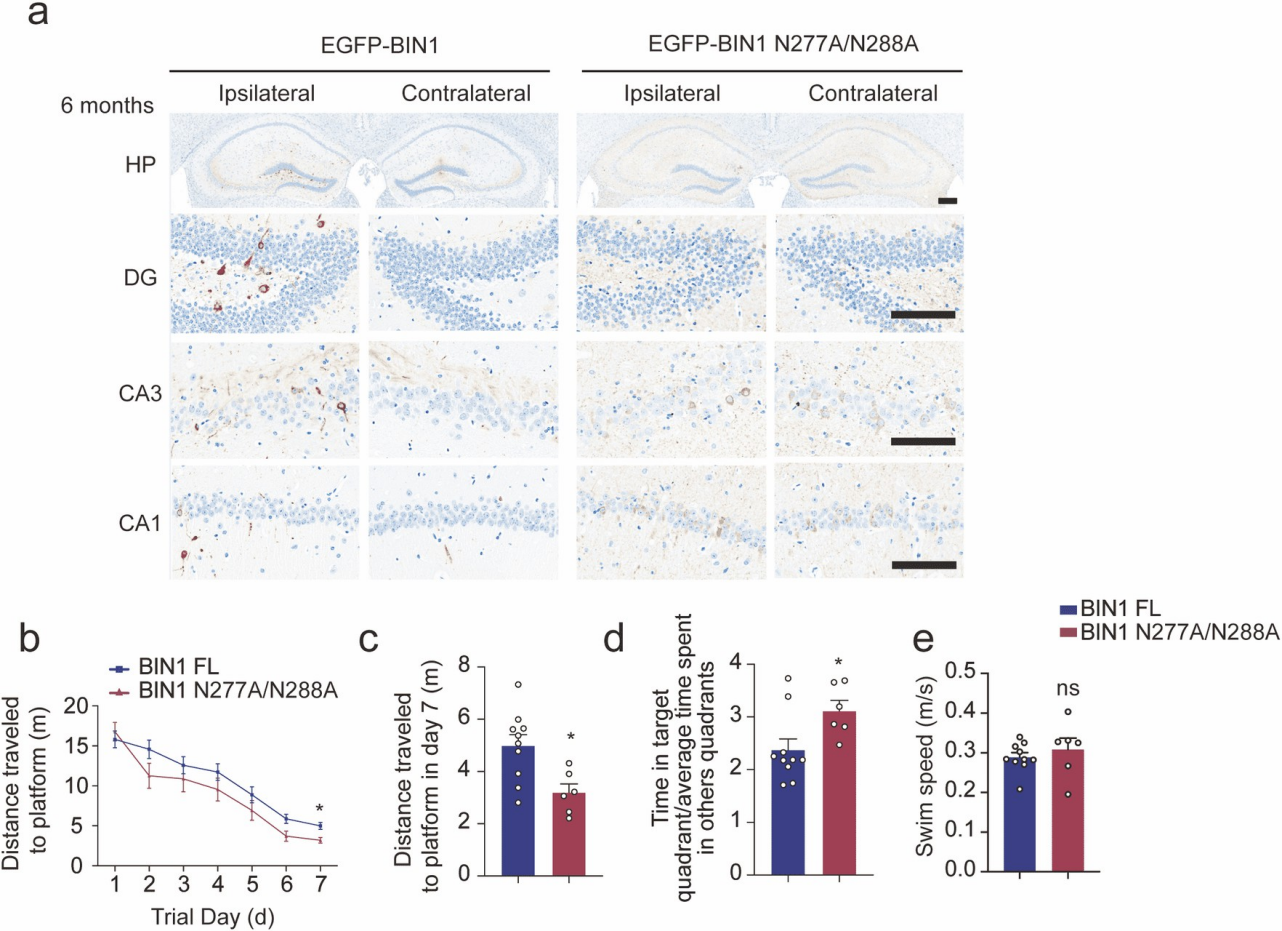
Uncleavable BIN1 inhibits the propagation of tau pathology. (**a**) AT8 immunostaining of the DG, CA3, and CA1 areas of the HP in tau P301S mice 6 months after the injection of a mixture of K18 fibrils and AAVs encoding wild-type or N277A/N288A mutant BIN1. Scale bar of the whole HP, 280 μm; scale bar of DG, CA3, and CA1, 140 μm. (**b**, **c**) Morris water maze analysis of the distance traveled to the platform (**b**) and the distance traveled to the platform on day 7 (**c**) (mean ± SEM; *n* = 6–10 mice per group; *P < 0.05, Student *t* test). (**d**) Probe trial of the Morris water maze test analyzed as time spent in the target quadrant versus the average of time spent in other quadrants (mean ± SEM; *n* = 6–10 mice per group; **P < 0.01, Student *t* test). (**e**) Swim speed of mice injected with AAVs encoding EGFP, EGFP-BIN1 FL, and EGFP-BIN1 N277A/N288A (mean ± SEM; *n* = 6–10 mice per group, Student *t* test). Source data can be found in S1 Data. AAV, adeno-associated virus; BIN1, bridging integrator 1; DG, dentate gyrus; EGFP, enhanced green fluorescent protein; FL, full-length; HP, hippocampus.

To verify the role of legumain-mediated BIN1 fragmentation in tau propagation, we injected AAVs encoding wild-type BIN1 or N277A/N288A mutant BIN1 that cannot be cleaved by legumain, together with K18 fibrils into the DG area of 2-month-old tau P301S mice. One month later, the severity of tau pathology was similar in both groups (S11A Fig). However, 2 months after injection, tau pathology was observed in the ipsilateral CA1 area in mice expressing wild-type BIN1 but not in mice expressing the uncleavable BIN1 (S11B Fig). Six months after injection, the ipsilateral DG, CA3, and CA1 areas in mice overexpressing wild-type BIN1 displayed more tau pathology than those in mice expressing the uncleavable BIN1 (Figs 7A and S12).

Electron microscopy and Golgi staining indicated that mice injected with uncleavable BIN1 showed a higher density of hippocampal synapses and dendritic spines than mice injected with uncleavable BIN1 (S12A–S12D Fig). In addition, in the water maze test, mice overexpressing uncleavable BIN1 traveled less distance to find the platform during the training phase than mice expressing wild-type BIN1 (Fig 7B and 7C). In the probe trial, the mice expressing mutant BIN1 spent more time in the target quadrant (Fig 7D). The swimming speeds of all mice were comparable (Fig 7E). These results indicate that blocking the cleavage of BIN1 by legumain alleviates the spreading of tau pathology in a mouse model of tauopathy.

### Blocking the fragmentation of endogenous BIN1 inhibits tau pathology propagation in vivo

**Fig 8:**
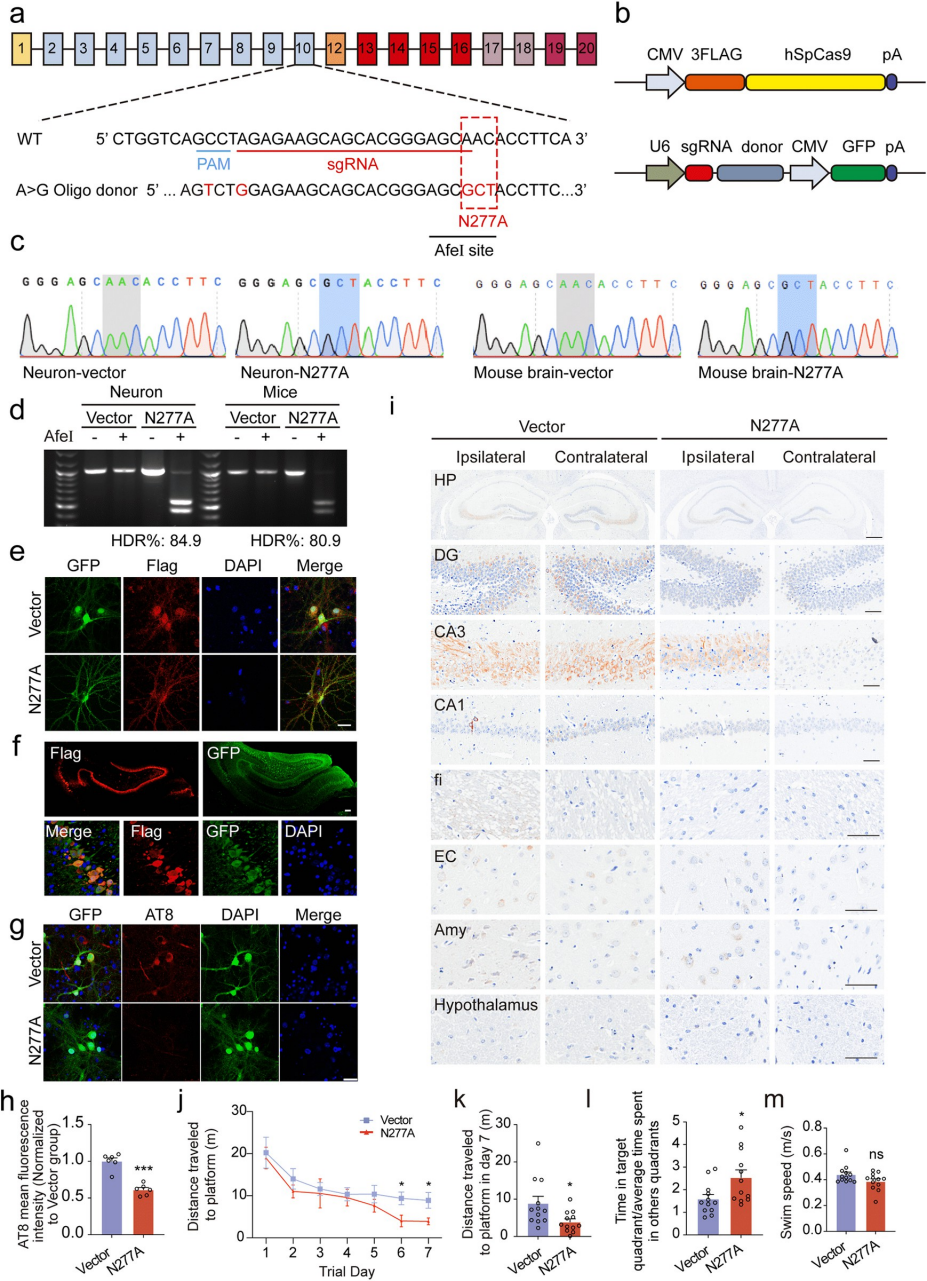
Blocking the cleavage of endogenous BIN1 attenuates the propagation of tau pathology in vivo. (**a**) Schematic diagram of the strategy to generate the BIN1^N277A^ mutation. The PAM (blue) and sgRNA (red) sequences are shown. The mutant site is indicated in the red dotted box. (**b**) Schematic of AAVs used for genome editing. (**c**) Sanger sequencing showing the point mutation in cultured neurons and mouse brain infected with the AAVs in (**b**) or control AAVs (vector). (**d**) AfeI enzyme digestion of DNA extracted from cultured neurons or mouse brains infected with the AAVs in (**b**) or control AAVs (vector). The HDR efficiency is indicated as the percentage of the cleaved fragments. (**e**) The immunostaining of anti-flag and anti-GFP in cultured neurons. Scale bar, 25 μm. (**f**) The immunostaining of anti-flag and anti-GFP in the HP of tau P301S mice. Scale bar, 100 μm. (**g**, **h**) AT8 immunostaining and quantification of cultured neurons of tau P301S mice infected with the AAVs in (**b**) or control AAVs (vector). Scale bar, 25 μm (mean ± SD; *n* = 6 mice per group; ***P < 0.001, Student *t* test). (**i**) AT8 immunostaining of DG, CA3, CA1, fi, EC, amygdala, and hypothalamus area of HP in tau P301S mice 2 months after the injection with the AAVs in (**b**) or control AAVs (vector). Scale bar of the whole HP, 500 μm; scale bar of other regions, 50 μm. (**j**, **k**) Morris water maze analysis as distance traveled to the platform (**j**) and distance traveled to the platform in day 7 (**k**) (mean ± SEM; *n* = 12 mice per group; **P < 0.01, Student *t* test). (**l**) Probe trial of Morris water maze test analyzed as time spent in the target quadrant versus the average of time spent in other quadrants (mean ± SEM; *n* = 12 mice per group; *P < 0.05, Student *t* test). (**m**) Swimming speed (mean ± SEM; *n* = 12 mice per group, Student *t* test). Source data can be found in S1 Data. AAV, adeno-associated virus; BIN1, bridging integrator 1; DG, dentate gyrus; EC, entorhinal cortex; fi, fimbria; GFP, green fluorescent protein; HDR, homology-directed repair; HP, hippocampus; PAM, protospacer adjacent motif; sgRNA, single guide RNA; WT, wild-type.

To demonstrate the physiological role of legumain-mediated fragmentation of endogenous BIN1 in the propagation of tau pathology, we used the CRISPR/Cas9 gene-editing system to introduce an N277A point mutation into endogenous BIN1 in primary neurons and the hippocampus of tau P301S mice (Fig 8A and 8B, S1 Table). Infections of the Cas9 components and donor templates successfully introduced a BIN1^N277A^ mutation in primary neurons and tau P301S mice (Fig 8C). The mutation creates a novel restriction enzyme site, the AfeI site, which can be used to detect the homology-directed repair (HDR) efficiency (Fig 8A). The HDR efficiency was as high as 84.9% in neurons, and 80.9% in the mouse brain (Fig 8D). These results indicate that the sgRNA specifically targets the N277 site of BIN1. The expression of Cas9 and donor templates was detected in primary neurons and tau P301S mice infected with Flag-tagged Cas9 components and GFP-tagged donor templates (Fig 8E and 8F). The BIN1 N277A mutation significantly decreased the generation of BIN1 (1–277) in primary neurons and brain tissues of tau P301S mice (S14A–S14D Fig). Additionally, the BIN1 N277 mutation in primary neurons from tau P301S mice significantly restricted tau pathology induced by K18 fibrils (Fig 8G and 8H). Moreover, we found that the progression of tau pathology in tau P301S mice carrying the BIN1^N277A^ mutation was slower than that in control mice. Two months after injection with K18 fibrils, tau pathology progressed from the DG region to the ipsilateral CA3 and CA1 regions in tau P301S mice, while tau pathology was restricted to the DG region in mice bearing the BIN1^N277A^ mutation (Figs 8I and S15). Additionally, in the water maze test, mice carrying the BIN1^N277A^ mutation traveled less distance to find the platform during the training phase than control mice (Fig 8J and 8K). In the probe test, mice carrying the BIN1^N277A^ mutation spent more time in the target quadrant than the control mice (Fig 8L). The swimming speeds of all mice were comparable (Fig 8M). These results demonstrate that blocking legumain-mediated cleavage of endogenous BIN1 alleviates the propagation of tau pathology in tau P301S mice.

## Discussion

In the present study, we found that BIN1 is a physiological substrate of legumain. Under physiological conditions, legumain is localized in the lysosome, whereas it leaks into the cytoplasm under pathological conditions [23,24]. The lysosomal leakage creates opportunities for the interaction between legumain and BIN1. Cleavage of BIN1 by legumain generates 2 N-terminal fragments, BIN1 (1–277) and BIN1 (1–288). The former is the major fragment in the brains of AD patients and tau P301S mice. It promotes CME and facilitates the uptake of tau aggregates. Furthermore, BIN1 (1–277) interacts with tau fibrils and accelerates tau assembly. BIN1 (1–277) also promotes the propagation of tau pathology in tau P301S mice and induces learning and memory impairments. Blocking the fragmentation of endogenous BIN1 by legumain ameliorates the spreading of tau pathology and attenuates behavioral deficits in tau P301S mice. Overall, these results demonstrate that the legumain-derived BIN1 (1–277) fragment promotes the assembly and propagation of tau.

There are at least 10 different isoforms of BIN1 [10, 11]. Isoform 1 is the longest isoform of BIN1 and is specifically expressed in neurons. Isoform 9 is mainly expressed in oligodendrocytes. Isoforms 6, 9, 10, and 12 are mainly expressed in microglia, while isoforms 2 and 3 are mainly expressed in astrocytes and neurons [10,11,25]. Here, we focused on BIN1 isoform 1 to illustrate the function of BIN1 and its fragments in neurons. The legumain cleavage sites at N277 and N288 are conserved among these isoforms. Thus, the isoforms expressed in glia can also be cleaved by legumain. We detected the N277 fragment in both neurons and glia (S1E Fig). The role of legumain-cleaved BIN1 fragments in glial cells deserves further research.

The transmission of pathological tau assemblies has been extensively studied. Endocytosis mediates the uptake of tau fibrils into recipient cells. Multiple forms of endocytosis have been reported, including bulk endocytosis [7], adsorptive endocytosis [6], micropinocytosis [26], and CME [22]. While clathrin inhibitors may not affect the uptake of tau aggregates [7,26], inhibition of dynamin, a protein involved in CME, has been shown to attenuate tau aggregate uptake [7,22]. BIN1 sequesters dynamin, thereby inhibiting CME and the propagation of tau [21]. Here, we confirmed that BIN1 inhibits CME and enlarges the puncta size of dynamin (Fig 4). However, legumain-generated BIN1 (1–277) promotes CME and facilitates the uptake of tau fibrils. BIN1 (1–277) lacks the SH3 domain, which is needed to mediate its interaction with dynamin, thus it fails to increase the size of dynamin puncta, as full-length BIN1 does in neurons. Rab5 is an early endosome marker. Previous studies reported that BIN1 binds RIN3 and competitively inhibits the binding of Rab5 and RIN3 [22]. However, BIN1 (1–277) lacks the domain that interacts with RIN3 [27], which may explain why BIN1 (1–277) loses the ability to restrict CME. However, BIN1 (1–277) contains the BAR domain, which is responsible for recruiting dynamin in the presence of PtdIns(4,5)P_2_ [28]. The ability to recruit dynamin may account for the promoting effect of BIN (1–277) on CME. CEM may act as a double-edged sword. On the one hand, blocking CEM reduces tau propagation [22]. On the other hand, shutting off CME may halt synaptic vesicle recycling and lead to neuronal injury. It is conceivable that BIN1 plays a beneficial role in preventing tau propagation by restricting the CME process, but compromised endocytosis may alter synaptic vesicle recycling [29]. However, BIN1 has also been reported to regulate the exocytosis of synaptic vesicles [30]. Loss of BIN1 reduces the release probability of vesicles [30]. BIN1 fragments generated by legumain may lose the normal functions of full-length BIN1 to regulate endocytosis and synaptic vesicle recycling.

Expression of BIN1 (1–277) in HEK293 cells stably transfected with soluble tau-RD promoted the formation of tau inclusions. The facilitatory effect of BIN1 (1–277) on tau aggregation was also confirmed by an in vitro aggregation assay. The assembly of tau K18 includes a lag phase, a growth phase, and a final plateau regime [31,32]. Many conditions have been demonstrated to accelerate the aggregation of tau, such as mutations that destabilize the native form [33,34], posttranslational modifications [35], and proteolytic cleavage of tau [17]. Additionally, preformed seeds, including homologous tau fibrils and heterologous α-synuclein fibrils [36], can substantially shorten the lag time of tau aggregation. Here, we identified a novel factor, BIN1 (1–277) generated by legumain, which accelerates the assembly of tau. Additionally, the structure of the K18 fibrils in the presence of BIN1 (1–277) differs from that of fibrils comprised of K18 alone. The former displays a paired arrangement with longer fibrils, while the latter shows a single straight arrangement with shorter fibrils.

Tau P301S transgenic mice are widely used as a mouse model of tauopathy. The mice develop tau pathology, synaptic dysfunction, and behavioral impairments, which are suitable for studying the role of BIN1 fragments in tau propagation. The results in the study may reflect a common mechanism in tauopathies. Exogenous tau fibrils have been demonstrated to induce the propagation of tau pathology along with neuronal connections in tau P301S mice [4]. Six months after the injection of K18 fibrils into the brain, tau pathology propagated from the ipsilateral to the contralateral region [37]. However, the detailed mechanisms underlying the spreading of tau pathology are not clear. In our study, we injected a mixture of K18 fibrils and AAV-BIN1, BIN1 (1–277), or BIN1 (278–594) into the hippocampal DG and monitored the propagation of tau pathology (Fig 6). Compared with that in mice expressing EGFP, the spreading of tau pathology was attenuated in mice expressing full-length BIN1. However, the mice expressing BIN1 (1–277) exhibited the fastest tau propagation among all the mice analyzed. This phenomenon is consistent with the enhancing role of BIN1 (1–277) in tau propagation and assembly in vitro. We also found that BIN1 (1–277) fragment promotes the synaptic loss in tau P301S. However, the effect of BIN1 fragments on synaptic function and its mechanism is still unclear. One of the limitations of this study is the lack of data on the effects of BIN1 fragments on synaptic function.

To identify whether blocking the cleavage of BIN1 by legumain could ameliorate AD pathology, we mutated amino acid 277 of endogenous BIN1 from N to A in primary neurons and adult tau P301S mice using the CRISPR/Cas9-mediated gene-editing tool. Our results showed that tau pathology was attenuated by the N277A mutation of endogenous BIN1 in tau P301S mice (Fig 8). These results indicate that legumain-mediated fragmentation of BIN1 is crucial for the progression of tau pathology in AD. Blocking the cleavage of BIN1 by legumain may be an effective therapeutic strategy for AD.

## Materials and methods

### Ethics statement

The animal protocol was reviewed and approved by the Animal Care and Use Committee of Renmin Hospital of Wuhan University (20180813). The study including human tissue samples was approved by the Clinical Ethics Committee of Renmin Hospital of Wuhan University (WDRY2021-K028). Informed consent was obtained from all patients.

### Reagents

Primary antibodies against the following targets were used: GST (Proteintech, 66001-2-Ig, 1:5,000), EGFP-HRP (Proteintech, HRP-66002, 1:5,000), His (Proteintech, 66005-1-Ig, 1:5,000), GAPDH (Proteintech, 60004-1-Ig, 1:5,000), BIN1 (Santa Cruz, sc-23918, 1:1,000), HA (Proteintech, 51064-2-AP, 1:1,000), AT8 (Thermo, MN1020, 1:1,000), AT100 (Thermo, MN1000, 1:1,000), and tau5 (Thermo, MA5-12805, 1:1,000). Recombinant legumain was purchased from Sino Biological (Beijing, China). The TUNEL in situ cell death detection kit was from Roche (Indianapolis, IN). Transferrin Alexa Fluor 546 (T23364) and FM 4–64 (T13320) were purchased from Thermo Fisher.

### Mice

Wild-type C57BL/6J mice and Tau P301S mice (line PS19) were obtained from the Jackson Laboratory (stock numbers: 000664 and 008169, respectively). Only male mice were used in the study. Animal care and handling were carried out according to the Declaration of Helsinki and the guidelines of Renmin Hospital, Wuhan University. The mice were housed in a temperature (22.2 to 25.7°C) and humidity (30% to 70%) controlled room with a 12:12-h light:dark cycle. All animals were allowed ad libitum access to water and food (5001 rodent chow, LabDiet). The sample size was determined by Power and Precision (Biostat). Animals were randomly allocated to different groups. Investigators were blinded to the group assignment during the animal experiments.

### Human tissue samples

Postmortem brain samples from AD cases and age-matched controls were dissected from the frontal cortex in frozen hemisphere slices, which were from the Emory Alzheimer’s Disease Research Center and the Brain Bank at Xiangya School of Medicine [38,39]. The disease state, postmortem interval (PMI), age of death, sex, and apolipoprotein (ApoE) genotype are listed in S1 Table. The pathological diagnosis of AD was confirmed by the assessment of amyloid plaques and neurofibrillary tangles in the brain according to the NIH guideline [40]. For immunofluorescence staining of human brain tissue, the brain sections were incubated in 0.1% Sudan Black B (SSB) and 70% ethanol for 20 min to eliminate the autofluorescence signal.

### Cell lines and plasmid transfection

HEK293 cells and COS-7 cells were obtained from the ATCC and tested for mycoplasma contamination before use. Clone 1 and clone 9 cells were obtained from Dr. Marc I. Diamond [19]. Clone 1 cells stably express tau RD with a YFP tag, while clone 9 cells stably carry insoluble aggregates of YFP-tau RD. We used the following plasmids: EGFP-BIN1 (isoform1, human brain clone) was a gift from Dr. De Camilli P (Yale University School of Medicine) [41]; myc-legumain and myc-legumain C189S plasmids were the same as previously described [23]; EGFP-BIN1 point mutations were introduced using primers with the desired mutations. BIN1 FL, BIN1 (1–277), and EGFP-BIN1 (278–594) constructs were generated by PCR amplification of the full-length BIN1 (isoform 1, 1–594 aa), BIN1 (1–277 aa), or BIN1 (278–594 aa) cDNA. The PCR products were subcloned into the pEGFP-C2 vector or HA vector. HEK293 cells were transfected with these plasmids using polyethylenimine (PEI).

### In vitro BIN1 cleavage assay and legumain activity assay

BIN1 cleavage assays and legumain activity assays were performed as described previously [17]. Briefly, HEK293 cell lysates overexpressing EGFP-BIN1 were incubated with recombinant legumain (5 μg ml^−1^) at pH 7.4 or pH 6.0 in legumain buffer (50 mM sodium citrate, 5 mM DTT, 0.1% CHAPS and pH 5.5, 0.5% Triton X-100) at 37°C for different time points. To measure the cleavage of purified BIN1 by legumain, EGFP-BIN1 was immunoprecipitated by GFP antibody and protein A/G beads (Santa Cruz Biotechnology) and then incubated with recombinant legumain in legumain buffer at pH 6.0. The samples were then boiled in the loading buffer and analyzed by immunoblotting. For the legumain activity assay, the cell lysates were incubated in 200 μl assay buffer (20 mM citric acid, 60 mM Na_2_HPO_4_, 1 mM EDTA, 0.1% CHAPS, and 1 mM DTT (pH 6.0)) containing 20 μM legumain substrate Z-Ala-Ala-Asn-AMC (Bachem). AMC released by enzymatic cleavage was quantified by measuring at 460 nm in a fluorescence plate reader at 37°C for 1 h in the kinetic model of 5 min.

### Western blot analysis

The mouse brain tissue or human tissue samples were lysed in lysis buffer (50 mM Tris (pH 7.4), 40 mM NaCl, 1 mM EDTA, 0.5% Triton X-100, 1.5 mM Na_3_VO_4_, 50 mM NaF, 10 mM sodium pyrophosphate, and 10 mM sodium β-glycerophosphate, supplemented with protease inhibitor cocktail) and centrifuged for 15 min at 16,000*g*. The supernatant was boiled in SDS loading buffer. After SDS-PAGE, the samples were transferred to a nitrocellulose membrane. The membranes were incubated with primary antibodies overnight at 4°C, washed 3 times in PBST, and incubated with horseradish peroxidase (HRP)-conjugated secondary antibodies. The signals were developed using enhanced chemiluminescent (ECL) substrates.

### K18 uptake assay

Primary neurons were incubated with 250 nM His-K18 fibrils for different time points. The cells were subsequently fixed in 4% paraformaldehyde for 10 min followed by immunofluorescence staining. The intensity of internalized K18 in a single cell was measured using ImageJ software (*n* = 60 cells per group). To investigate the degradation of K18 fibrils, primary neurons were incubated with 250 nM His-K18 fibrils for 30 min. Then, free fibrils were washed away using PBS. The levels of K18 fibrils in the neurons were analyzed by immunostaining at 30 min, 360 min, or 720 min after incubation.

### Cell coculture system

COS-7 cells were transfected with HA-Vector, HA-BIN1-FL, HA-BIN1 (1–277), and HA-BIN1 (278–594) plasmids. HEK293 cells that contain tau inclusions (clone 9) were used as donor cells. The donor cells and COS-7 cells were trypsinized for 5 min at 37°C, resuspended in culture medium, and plated on 6-well plates. After being cocultured for 24 h, the cells were fixed and immunostained with an anti-HA antibody.

### Transferrin uptake assay

The neurons were infected with AAVs encoding EGFP, EGFP-BIN1, EGFP-BIN1 (1–277), or EGFP-BIN1 (278–594). Seven days later, Alexa Fluor 546-conjugated transferrin (Invitrogen, 25 μg ml^−1^) was added to the medium and incubated for 30 min at 37°C. The cells were washed in PBS and fixed in 4% paraformaldehyde. The fluorescence intensity of internalized transferrin was measured using ImageJ software (*n* = 30 cells per group).

### FM 4–64 dye uptake assay

FM 4–64 dye uptake assay was performed as described previously [42]. Briefly, the neurons were incubated in high-K^+^ Tyrode’s solution (NaCl 49.7 mM, KCl 90 mM, MgCl_2_ 1 mM, CaCl_2_ 1.8 mM, Na_2_HPO_4_ 0.2 mM, NaHCO_3_ 12 mM, D-Glucose 5.5 mM) with 10 μM FM 4–64 dye for 1 to 2 min to load the synaptic vesicles with the FM 4–64 dye. The cells were then switched to normal Tyrode’s solution (NaCl 137 mM, KCl 2.7 mM, MgCl_2_ 1 mM, CaCl_2_ 1.8 mM, Na_2_HPO_4_ 0.2 mM, NaHCO_3_ 12 mM, D-Glucose 5.5 mM) in the presence of FM 4–64 for 15 to 20 min. The staining solution was washed with a dye-free solution. High-K^+^ Tyrode’s solution was added to induce unloading. Neurons were rinsed 3 times for continued imaging. The FM 4–64 fluorescence intensity of boutons was measured by ImageJ software (*n* = 30 cells per group).

### His pull-down assay

His-K18 monomer, oligomers, or fibrils were incubated with Ni-NTA Agarose for 4 h at 37°C. After washing 4 times with PBS, the beads were incubated with HEK293 cell lysates overexpressing EGFP, EGFP-BIN1, and EGFP-BIN1 (1–277) overnight at 4°C. The beads were washed 4 times with PBS, boiled in SDS loading buffer, and analyzed by immunoblotting.

### Immunostaining

To investigate the presence of legumain-cleaved BIN1 in human AD brain sections, the sections were treated with 0.3% H_2_O_2_ for 10 min and washed 3 times in PBS. Then, sections were blocked in PBS with 5% BSA and 0.3% Triton X-100 for 30 min and incubated with anti-BIN1 N277 and N288 (generated and verified as described in the main text, 1:1,000). To detect p-tau, free-floating 30-μm-thick brain sections were incubated with AT8 or AT100 overnight at 4°C. The signal was developed using a Histostain-SP kit (Invitrogen).

### K18 expression, purification, and aggregation

Human tau fragment containing the 4 microtubule-binding repeat domains (K18) was fused with an N-terminal His-tag and subcloned into the pRK172 vector. Recombinant His-K18 was purified as described previously [36]. For in vitro K18 fibril production, a solution of 1 mg/mL K18, 12.5 μM low–molecular weight heparin, and 2 mM DTT in PBS buffer was incubated at 37°C for 6 to 12 h.

The aggregation of tau was quantified by incubating with 50 μM ThS and measuring the fluorescence with an excitation wavelength of 440 nm and an emission wavelength of 510 nm. The overall appearance of the fibrils was visualized by negative stain electron microscopy.

### Co-sedimentation assay and mass spectrometry

K18 (1 mg/mL) was mixed with N277 peptide (0.5 mg/mL) in PBS. Low–molecular weight heparin (12.5 μM) and DTT (2 mM) were added to the mixture and incubated at 37°C for 12 h. The fibrils were centrifuged at 100,000 × *g* for 1 h. The pellet was washed with PBS and centrifuged again at 100,000 × *g* for 30 min. The pellets were resuspended and detected by MS. The data were collected using the Q Exactive HF mass spectrometer in series with the UltiMate 3000 RSLCnano liquid phase liquid chromatography–mass spectrometry system. The peptide sample was dissolved in the loading buffer, inhaled by an automatic sampler, and then passed through the analysis column (75 μM * 25 cm, C18, 1.9 μm. 120 Å) for separation. Two mobile phases (mobile phase A: 0.1% formal acid, 3% DMSO; and mobile phase B: 0.1% formal acid, 3% DMSO, and 80% ACN) were used to establish an analytical gradient. The flow rate of the liquid phase was set to 300 nL/min. Mass spectrometry collected data in DDA mode, with each scan cycle consisting of one MS full scan (R = 60 K, AGC = 3e6, max IT = 25 ms, scan range = 350 to 1,500 m/z), followed by 20 MS/MS scans (R = 15 K, AGC = 1e5, max IT = 50 ms). The HCD collision energy was set to 27. The screening window for the fourth level pole was set to 1.4 Da. The dynamic exclusion time for ion repeat collection was set to 24 s. Mass spectrometry data were retrieved using MaxQuant (V1.6.6) software using Andromeda as the database retrieval algorithm. The database used for retrieval was the Human Proteome Reference Database in UniProt (2022-03-29, containing 20377 protein sequences).

### Generation of antibodies that specifically recognize the legumain-generated BIN1 fragments (anti-BIN1 N277 and anti-N288 antibodies)

The anti-BIN1 N277 and N288 antibodies were generated by immunizing rabbits with the peptide Ac-CGLEKQHGSN-OH (anti-BIN1 N277) or Ac-CTVKAQPSDN-OH (anti-BIN1 N288), respectively. The antiserum was pooled, and the titers against the immunizing peptide were determined by ELISA. The maximal dilution giving a positive response with the chromogenic substrate for horseradish peroxidase was 1:512,000. The immunoactivity of the antiserum was further confirmed by western blotting and immunohistochemistry.

### ELISA quantification of the BIN1 (1–277) fragment and p-Tau in brain lysates

The 96-well Nunc-Immuno MaxSorp plates (VWR Cat. # 62409–024) were coated with anti-BIN1 (1–277) antibody that was diluted at 1:2,000 in PBS (100 μl/well). After being incubated overnight at 4°C, the plates were washed with PBS/Tween-20 (PBST; 0.5%, v/v) and then blocked using 2% BSA in PBST for 2 h at room temperature. The brain tissue lysates were diluted 1:1,000 and added to the plates (100 μl/well). The plates were incubated overnight at 4°C. After washing, the detection antibody (anti-BIN1, 1:1,000) was added and incubated for 4 h at 4°C. Then, the anti-mouse HRP-conjugated secondary antibody (Thermo Fisher, 1:5,000) was added and incubated for 2 h at room temperature. After washing, the TMB substrate solution (Sigma-Aldrich, CL07) was added to each well and incubated at 37°C for 20 min. The reaction was stopped by adding 3 N HCl, and the values of each well were recorded using a microplate reader at 450 nm. The concentrations of BIN1 (1–277) were calculated using the standard curve generated by purified BIN1 (1–277). The concentrations of p-Tau181 in the brain lysates were determined using the Tau (Phospho) [pT181] human ELISA kit (Thermo Fisher, # KHO0631).

### Primary neuron cultures

Primary rat cortical neurons were cultured as previously described [17]. Eighteen-day gestational mice were killed by cervical dislocation. After removing the meninges and blood vessels, the cerebral cortex was isolated and cut into pieces, resuspended in 3 mL medium (6% horse serum, 6% fetal bovine serum, 1% penicillin–streptomycin, and 1% glutamine in Dulbecco’s Modified Eagle Medium), and centrifuged at 1,000 rpm at 4°C for 5 min. The cell pellet was resuspended in the same medium and centrifuged at 1,000 rpm at 4°C for 5 min again. The cell pellet was resuspended and seeded into polylysine-coated 12-well plates. The cellular density was approximately 2.0 ×10^5^ cells per well in 12-well plates. The culture medium was changed to neuronal medium (2% B27 supplement, 1% penicillin–streptomycin, 1% glutamine in Neurobasal media) after culturing for 4 h. At 2 d in vitro (DIV2), cytarabine (10 μM final concentration) was added to the culture for 6 h to suppress the growth of glial cells. The purity of neurons was analyzed by immunofluorescence staining using neuronal and glial markers. AAVs encoding full-length BIN1, BIN1 (1–277), or BIN1 (278–594) were added to the medium of DIV5 neurons. The neurons were fixed on DIV14, permeabilized, and immunostained with AT8, AT100, and TUNEL. The sections were covered with a glass cover using a mounting solution and examined under a fluorescence microscope (Olympus).

### Screening of guide RNAs and vector production

The guide RNA was designed by visual inspection of the sequences according to the requirements of the NGG motif and spanning the BIN1 N277 site. The sgRNA and the donor sequence were inserted into the pAAV-U6-CMV-EGFP-WPRE expression vector. The corresponding protospacer adjacent motif (PAM) site was mutated (silent mutations) to reduce the targeting of the DNA repair template by Cas9. The sgRNA sequence, the donor sequence, and the primer sequences for PCR amplification of genomic DNA (gDNA) are provided in S2 Table. The spCas9 was inserted in the pAAV-CMV-3×FLAG expression vector.

### AAV preparation and stereotaxic injection

AAV particles encoding full-length BIN1 (isoform 1), BIN1 (1–277), and BIN1 (278–594) driven by the human synapsin I promoter were prepared by Shumi Technologies (Wuhan, China). CRISPR/Cas9 gene-editing viruses, including pAAV-CMV-3xFLAG-spCas9 and pAAV-U6-spgRNA (Bin1)-donor (N277A)-CMV-EGFP-WPRE, were prepared by Obio Technology (Shanghai). The unilateral intracerebral injection was performed stereotactically at the following coordinates: posterior 2.5 mm, lateral 2.0 mm, and ventral 1.7 mm relative to bregma. A mix of 5 μg His-K18 fibrils and 250 nl AAV-GFP, AAV-GFP-BIN1, AAV-GFP-BIN1 (1–277), or AAV-GFP-BIN1 (278–594) containing 1 × 10 [9] vector genomes (vg) was injected into each site using a 10-μl glass syringe with a fixed needle. To test the effect of endogenous BIN1 N277 mutation on the spreading of tau pathology in vivo, a mix of 5 μg His-K18 fibrils, pAAV-CMV-3xFLAG-spCas9, and pAAV-U6-spgRNA (Bin1)-donor (N277A)-CMV-EGFP-WPRE (1 × 10 [9] vg) were injected into the hippocampus.

### Nucleic acid and protein analyses

The gDNA from cultured neurons or mouse brains infected with the AAVs was extracted and PCR amplified by high-fidelity DNA polymerase. Approximately 1,000 bp amplicons across the N277 of gDNA were generated and analyzed for targeting efficiency using Sanger sequencing and AfeI digestion. The N277A mutation creates a novel restriction enzyme site, the AfeI site (AGCGCT), which allows digestion by AfeI. The protein of infected neurons and hippocampus in tau P301S mice were extracted and detected by western blotting using anti-BIN1 antibody and anti-N277 antibody.

### Electron microscopy of synapses

Synaptic density was determined by electron microscopy as described previously [42]. Mice were anesthetized and perfused transcardially with 2% glutaraldehyde. The hippocampal slices were postfixed in cold 1% OsO4 for 1 h. Samples were prepared and examined using standard procedures. Ultrathin sections (90 nm) were stained with uranyl acetate and lead acetate and viewed at 100 kV in a JEOL 200CX electron microscope. Synapses were identified by the presence of synaptic vesicles and postsynaptic densities.

### Morris water maze test

Five-month-old tau P301S mice were trained with extra maze cues as described previously [17]. Each subject was tested in 4 trials per day for 7 consecutive days with a 15-min intertrial interval. If the subjects did not reach the platform within 60 s, they were manually guided to the platform. Following the 7 days of task acquisition, a probe trial was performed on day 7. The platform was removed, and the distance traveled in the target quadrant was measured over 90 s. All trials were analyzed for latency and swim speed using ANY-Maze software (San Diego Instruments).

### Statistical analyses

Statistical analysis was performed using either Student *t* test (two-group comparison) or one-way ANOVA followed by LSD post hoc test (more than 2 groups). Differences with *P* values less than 0.05 were considered significant.

## Supporting information

S1 Fig Verification of the anti-BIN1 N277 and anti-BIN1 N288 antibodies. (**a**) Western blots showing the fragmentation of GFP-BIN1 after incubation with legumain for 30, 45, and 60 min (left panel) or mouse brain lysates for 30 min (right panel). (**b**) Western blot showing the specificity of anti-BIN1 N277 and N288 antibodies (the uncropped figure of Fig 1A). (**c**) Immunohistochemistry showing the specificity of anti-BIN1 N277 and N288 antibodies. Preincubation of the antibodies with BIN1 (268–277) or BIN1 (279–287) peptide before immunohistochemistry blocked the signal. The staining was detected in WT mouse brain sections but not in legumain KO mouse brain sections. Scale bar, 100 μm. (**d**) The staining controls including omissions of the anti-N277 antibody, anti-N288 antibody, or secondary antibody in brain slices of WT mice, legumain^−/−^ mice, AD patients, or control subjects. Scale bar, 20 μm. (**e**) The levels of BIN1 (1–277) and BIN1 (1–288) in different brain cells, including neurons (MAP2), microglia (IBA1), astrocytes (GFAP), and oligodendrocytes (OLIG2). Scale bar, 25 μm. Source data can be found in S1 Raw Images. AD, Alzheimer’s disease; BIN1, bridging integrator 1; GFP, green fluorescent protein; KO, knockout; WB, western blot; WT, wild-type.
(TIF)

S2 Fig Relationship between the N277 fragment and tau pathology. (**a**, **b**) Western blot analysis and quantification of BIN1, BIN1 (1–277), and BIN1 (1–288) in young control subjects, old control subjects, and AD patients (mean ± SEM). *n* = 4 patients per group, one-way ANOVA, ***P < 0.001. (**c**) IF of BIN1 N277 fragments and AT8 in brain sections from AD patients with different Braak stages. Scale bar, 20 μm. (**d**, **e**) Quantification of AT8 and N277 immunostaining (mean ± SEM; one-way ANOVA, *n* = 6 sections from 3 patients). **P < 0.01, ***P < 0.001. (**f**) Correlation between the concentrations of BIN1 (1–277) and p-Tau181 as determined by ELISA (R-squared = 0.8841, *P* < 0.001). Source data can be found in S1 Data and S1 Raw Images. AD, Alzheimer’s disease; BIN1, bridging integrator 1; IF, immunofluorescence.
(TIF)

S3 Fig The expression of the BIN1 (1–277) fragment in tau P301S mice. (**a**, **b**) Double immunostaining of anti-N277 (red) (**a**) and anti-N288 (red) (**b**) with BIN1 (green) in brain sections from tau P301S mice at different ages. Scale bar, 20 μm. (**c**) Quantification of the immunoreactivity in (**a**) (mean ± SEM; one-way ANOVA, *n* = 6; **P < 0.01, ***P < 0.001 compared with 4-month-old mouse brain). (**d**) Quantification of immunoreactivity in (**b**) (mean ± SEM; one-way ANOVA, *n* = 6). (**e**) Western blot analysis of BIN1, BIN1 (1–277), and BIN1 (1–288) in tau P301S mouse brain. (**f**) Quantification of immunoreactivity in (**e**) (mean ± SEM; one-way ANOVA, *n* = 4; *P < 0.05, ***P < 0.001 compared with 2-month-old mouse brain). Source data can be found in S1 Data and S1 Raw Images. BIN1, bridging integrator 1; FL, full-length; WB, western blot.
(TIF)

S4 Fig Overexpression of BIN1 and its fragments does not affect neuronal viability. (**a**) Immunofluorescence staining of the neuronal marker MAP2, astrocyte marker GFAP, or microglial marker Iba1. Scale bar, 25 μm. (**b**) LDH release assay of neurons expressing full-length and fragmented BIN1 (mean ± SEM; one-way ANOVA, *n* = 5 independent experiments). Source data can be found in S1 Data. BIN1, bridging integrator 1; FL, full-length; GFP, green fluorescent protein; LDH, Lactate Dehydrogenase.
(TIF)

S5 Fig Degradation of K18 in neurons and interaction of BIN1 fragments with dynamin. (**a**) Neurons expressing BIN1, BIN1 (1–277), or BIN1 (278–594) were exposed to K18 fibrils and washed with PBS. The representative immunofluorescence images show the K18 signals in neurons at different time points (0, 30 min, 360 min, and 720 min) after washing. Scale bar, 10 μm. (**b**) Quantification of K18 signals in (**a**) (mean ± SEM *n* = 60 cells per group; ***P < 0.001). (**c**) His pull-down assay shows that dynamin interacts with BIN1 and BIN1 (278–594), but not BIN1 (1–277). Source data can be found in S1 Data and S1 Raw Images. BIN1, bridging integrator 1; FL, full-length; GFP, green fluorescent protein; GST, Glutathione S-Transferase; MW, molecular weight; WB, western blot.
(TIF)

S6 Fig Co-sedimentation assay and seeding activity assay of mixed fibrils. (**a**, **b**) MS/MS spectrum showing the identification of tau (**a**) and BIN1 N277 peptide (**b**). (**c**) HEK293 cells stably expressing tau RD-GFP were transduced with fibrils formed by K18 in the presence or absence of BIN1 N277 peptide, BIN1 N288 peptide, and BIN1 spanning peptide and permeabilized with 1% Triton X-100. The green dots indicate insoluble tau aggregates. Scale bar, 20 μm. (**h**) Quantification of the percentage of cells containing aggregates in cells. Data represent mean ± SEM of 4 independent experiments. *P < 0.05, ***P < 0.001. BIN1, bridging integrator 1; GFP, green fluorescent protein; RD, repeat domain.
(TIF)

S7 Fig The expression of BIN1 and its fragments in tau P301S mice. (**a**) Western blot showing the levels of BIN1 and BIN1 (1–277) in tau P301S mice. (**b**) Immunofluorescence showing that GFP-BIN1 fragments are mainly expressed in neurons, but not in astrocytes and microglia. Scale bar, 20 μm. Source data can be found in S1 Raw Images. BIN1, bridging integrator 1; FL, full-length; GFP, green fluorescent protein; MW, molecular weight.
(TIF)

S8 Fig Quantification of AT8 immunostaining in different brain regions of tau P301S mice at 6 months after injection with AAV-BIN1 FL or AAV-BIN1 fragments. Mean ± SEM; *n* = 7 mice per group, *P < 0.05, **P < 0.01. ***P < 0.001. Source data can be found in S1 Data. AAV, adeno-associated virus; BIN1, bridging integrator 1; DG, dentate gyrus; EC, entorhinal cortex; FL, full-length; Fi, fimbria; p-tau, phospho-tau.
(TIF)

S9 Fig BIN1 (1–277) promotes the propagation of tau pathology in tau P301S mice. (**a**, **b**) AT8 immunostaining of the DG, CA3, and CA1 areas of the HP in tau P301S mice at 1 month (**a**) and 2 months (**b**) after injection of a mixture of K18 fibrils and AAVs encoding BIN1 FL, (1–277), or (278–594). Scale bar of the whole HP, 280 μm; Scale bar of DG, CA3, and CA1, 80 μm. AAV, adeno-associated virus; BIN1, bridging integrator 1; DG, dentate gyrus; EGFP, enhanced green fluorescent protein; FL, full-length; HP, hippocampus.
(TIF)

S10 Fig BIN1 (1–277) promotes synaptic loss. (**a**) Electron microscopy of synapses. Arrows indicate synapses. Scale bar, 2 μm. (**b**) Quantification of synaptic density (mean ± SEM; *n* = 5 mice per group; *P < 0.05, ***P < 0.001, one-way ANOVA). (**c**) Golgi staining revealed the dendritic spines from the apical dendritic layer of the CA1 region. Scale bar, 2.5 μm. (**d**) Quantification of spine density (mean ± SEM; *n* = 5 mice per group; **P < 0.01, one-way ANOVA). Source data can be found in S1 Data. BIN1, bridging integrator 1; EGFP, xxxx; FL, xxxx.
(TIF)

S11 Fig Overexpression of BIN1 N277A/N288A inhibits the propagation of tau pathology in vivo. (**a**, **b**) AT8 immunostaining of DG, CA3, and CA1 areas of the HP in tau P301S mice at 1 month (**a**) and 2 months (**b**) after injection of a mix of K18 fibrils and AAVs encoding wild-type or N277A/N288A mutant BIN1. Scale bar of the whole HP, 280 μm; Scale bar of DG, CA3, and CA1, 140 μm. AAV, adeno-associated virus; BIN1, bridging integrator 1; DG, dentate gyrus; EGFP, xxxx; HP, hippocampus.
(TIF)

S12 Fig Quantification of AT8 immunostaining in different brain regions of tau P301S mice at 6 months after injection of AAV-BIN1 FL or AAV-BIN1 N277A/N288A. Mean ± SEM; *n* = 7 mice per group, *P < 0.05, **P < 0.01. ***P < 0.001. Source data can be found in S1 Data. AAV, adeno-associated virus; BIN1, bridging integrator 1; DG, dentate gyrus; FL, full-length; p-tau, phospho-tau.
(TIF)

S13 Fig BIN1 N277A/N288A attenuates synaptic loss induced by K18 fibrils. (**a**) Electron microscopy of synapses. Arrows indicate synapses. Scale bar, 2 μm. (**b**) Quantification of synaptic density (mean ± SEM; *n* = 5 mice per group; *P < 0.05, Student *t* test). (**c**) Golgi staining revealed the dendritic spines from the apical dendritic layer of the CA1 region. Scale bar, 2.5 μm. (**d**) Quantification of spine density (mean ± SEM; *n* = 5; *P < 0.05, Student *t* test). Source data can be found in S1 Data. BIN1, bridging integrator 1; EGFP, enhanced green fluorescent protein; FL, full-length.
(TIF)

S14 Fig BIN1^N277A^ mutation blocks the production of BIN1 (1–277). (**a**, **b**) The expression of BIN1 (1–277) in primary neurons infected with control AAVs (vector) or AAVs mediating the mutation (N277A). (**c**, **d**) The control AAVs (vector) or AAVs mediating the mutation (N277A) were injected into the left hippocampus of tau P301S mice. Western blot shows the expression of BIN1 (1–277) in brain lysates (mean ± SEM; one-way ANOVA, *n* = 6). *P < 0.05, **P < 0.01, ***P < 0.001. L, left; R, right. Source data can be found in S1 Data and S1 Raw Images. AAV, adeno-associated virus; BIN1, bridging integrator 1; FL, full-length; MW, molecular weight.
(TIF)

S15 Fig Quantification of AT8 immunostaining in different brain regions of tau P301S mice injected with AAV-BIN1 or AAV-BIN1^N277A^. Mean ± SEM; *n* = 7 mice per group, *P < 0.05, **P < 0.01. ***P < 0.001. Source data can be found in S1 Data. AAV, adeno-associated virus; BIN1, bridging integrator 1; DG, dentate gyrus; FL, xxxx; p-tau, phospho-tau.
(TIF)

S1 Table The neuropathological characteristics of human samples. (DOCX)

S2 Table The sequence of sgRNA, the donor sequence, and the primer sequences for PCR amplification of genomic DNA (gDNA). (DOCX)

S1 Raw images Original scan images. (PDF)

S1 Data Raw data. (XLSX)

## Abbreviations

AAV: adeno-associated virus
AD: Alzheimer’s disease
APP: amyloid precursor protein
AP2: adaptor protein 2
BIN1: bridging integrator 1
CA: cornu ammonis
CME: clathrin-mediated endocytosis
DG: dentate gyrus
EC: entorhinal cortex
EGFP: enhanced green fluorescent protein
FL: full-length
fi: fimbria
FM: FM 4-64 dye
gDNA: genomic DNA
GFP: green fluorescent protein
GST: Glutathione S-Transferase
GWAS: genome-wide association study
HA: human influenza hemagglutinin
HDR: homology-directed repair
HRP: horseradish peroxidase
LDH: Lactate Dehydrogenase
PAM: protospacer adjacent motif
PFC: prefrontal cortex
PMI: postmortem interval
p-tau: phospho-tau
RD: repeat domain
sgRNA: single guide RNA
ThS: thioflavin S
vg: vector genomes
YFP: yellow fluorescent protein
