# Supplementary figures and images for: Bridging integrator 1 fragment accelerates tau aggregation and propagation by enhancing clathrin-mediated endocytosis in mice
Source: PLoS Biol. 2024 Jan 11;22(1):e3002470. doi: 10.1371/journal.pbio.3002470 (PMC10783739; doi:10.1371/journal.pbio.3002470)

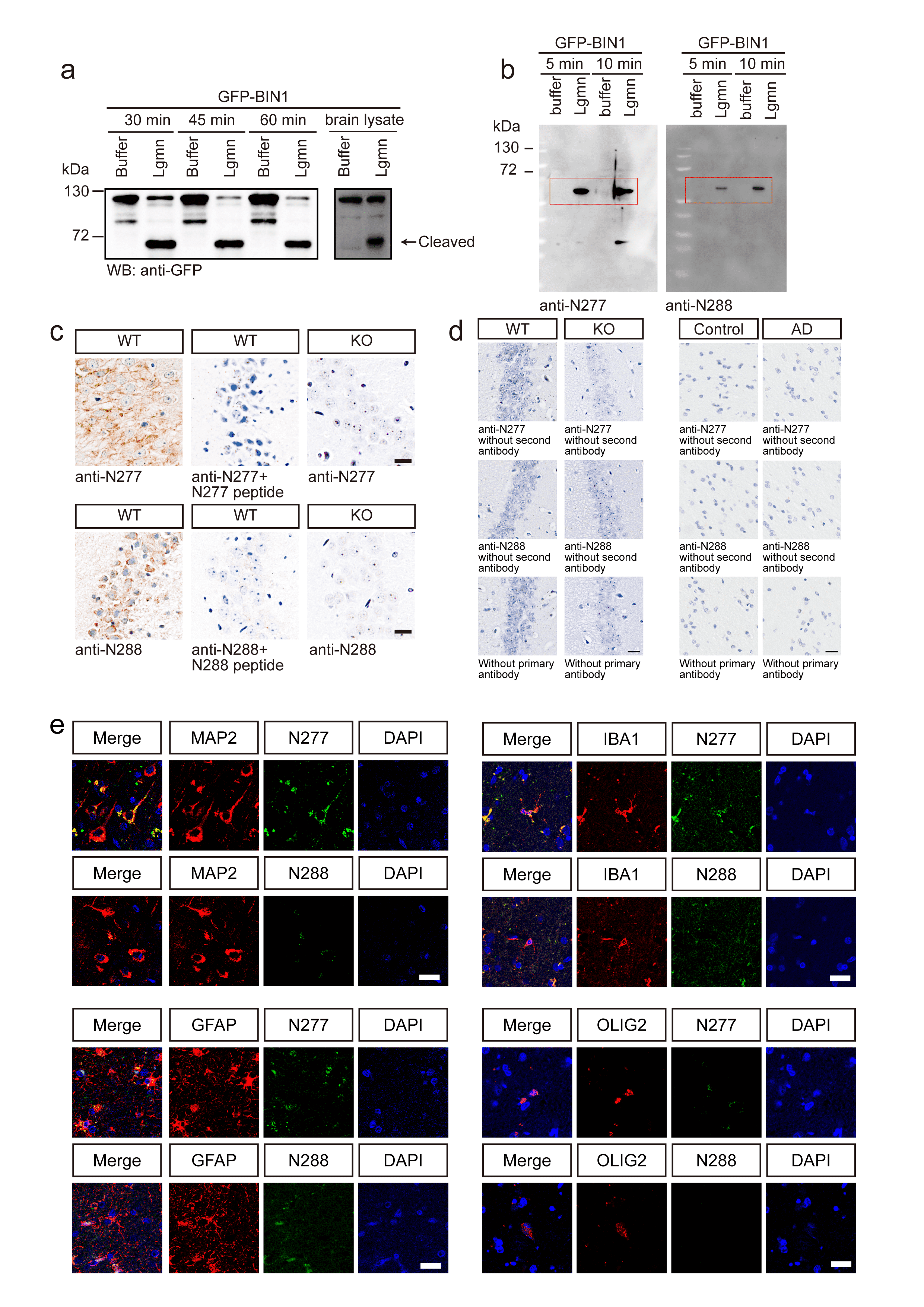

Supplement: S1 Fig — (a) Western blots showing the fragmentation of GFP-BIN1 after incubation with legumain for 30, 45, and 60 min (left panel) or mouse brain lysates for 30 min (right panel). (b) Western blot showing the specificity of anti-BIN1 N277 and N288 antibodies (the uncropped figure of Fig 1A). (c) Immunohistochemistry showing the specificity of anti-BIN1 N277 and N288 antibodies. Preincubation of the antibodies with BIN1 (268–277) or BIN1 (279–287) peptide before immunohistochemistry blocked the signal. The staining was detected in WT mouse brain sections but not in legumain KO mouse brain sections. Scale bar, 100 μm. (d) The staining controls including omissions of the anti-N277 antibody, anti-N288 antibody, or secondary antibody in brain slices of WT mice, legumain−/− mice, AD patients, or control subjects. Scale bar, 20 μm. (e) The levels of BIN1 (1–277) and BIN1 (1–288) in different brain cells, including neurons (MAP2), microglia (IBA1), astrocytes (GFAP), and oligodendrocytes (OLIG2). Scale bar, 25 μm. Source data can be found in S1 Raw Images. AD, Alzheimer’s disease; BIN1, bridging integrator 1; GFP, green fluorescent protein; KO, knockout; WB, western blot; WT, wild-type. (TIF) [file pbio.3002470.s001.tif]

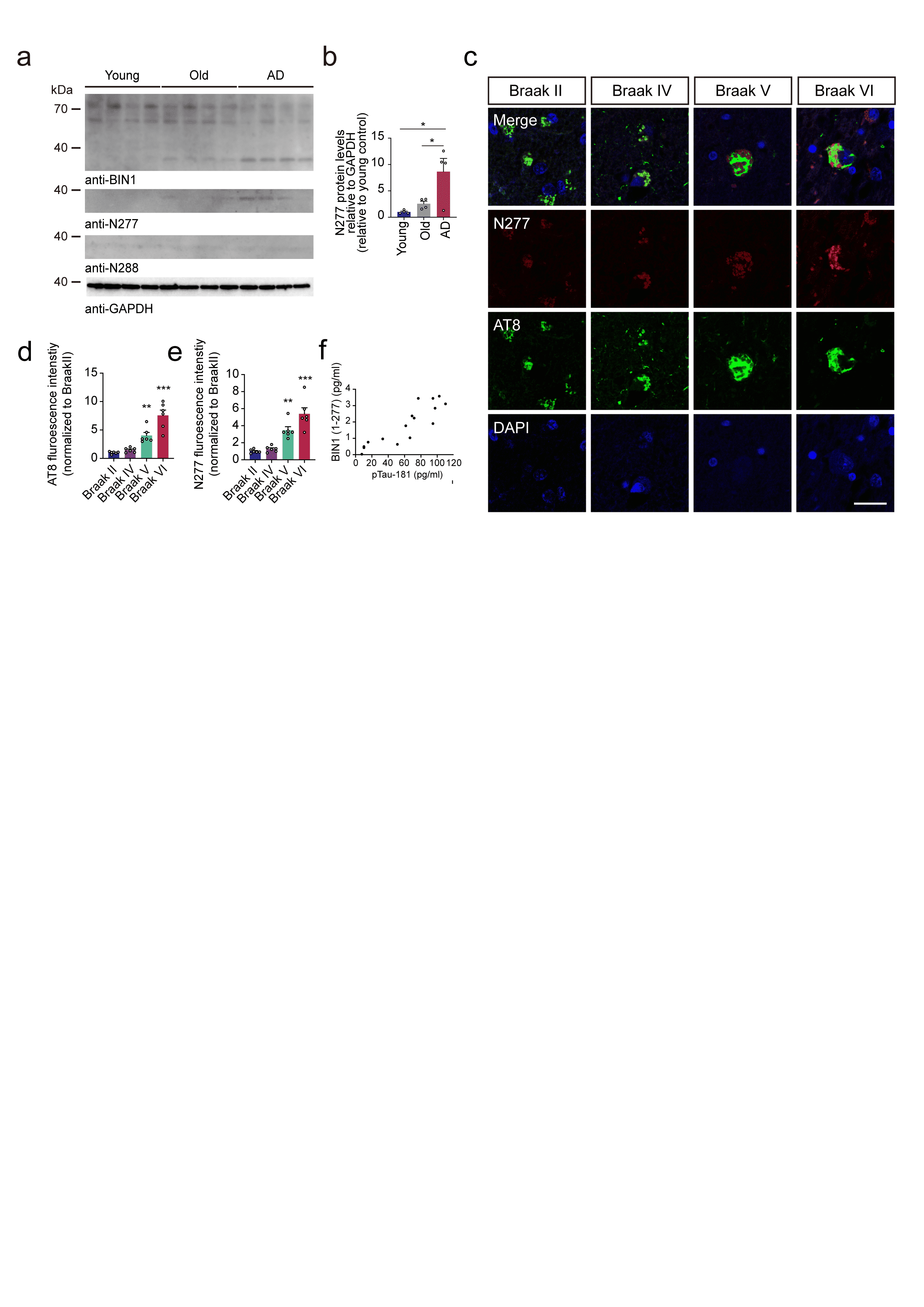

Supplement: S2 Fig — (a, b) Western blot analysis and quantification of BIN1, BIN1 (1–277), and BIN1 (1–288) in young control subjects, old control subjects, and AD patients (mean ± SEM). n = 4 patients per group, one-way ANOVA, ***P < 0.001. (c) IF of BIN1 N277 fragments and AT8 in brain sections from AD patients with different Braak stages. Scale bar, 20 μm. (d, e) Quantification of AT8 and N277 immunostaining (mean ± SEM; one-way ANOVA, n = 6 sections from 3 patients). **P < 0.01, ***P < 0.001. (f) Correlation between the concentrations of BIN1 (1–277) and p-Tau181 as determined by ELISA (R-squared = 0.8841, P < 0.001). Source data can be found in S1 Data and S1 Raw Images. AD, Alzheimer’s disease; BIN1, bridging integrator 1; IF, immunofluorescence. (TIF) [file pbio.3002470.s002.tif]

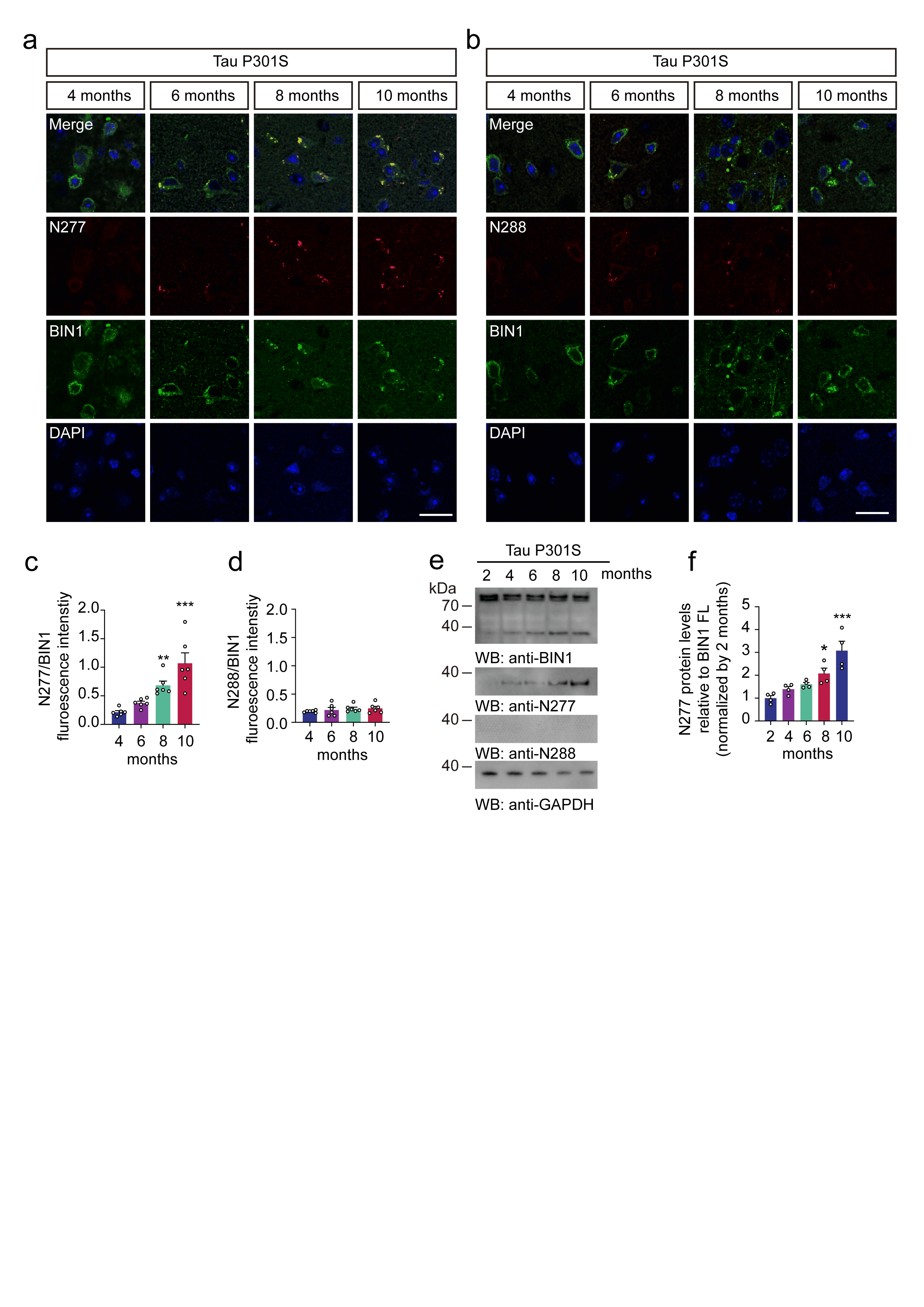

Supplement: S3 Fig — (a, b) Double immunostaining of anti-N277 (red) (a) and anti-N288 (red) (b) with BIN1 (green) in brain sections from tau P301S mice at different ages. Scale bar, 20 μm. (c) Quantification of the immunoreactivity in (a) (mean ± SEM; one-way ANOVA, n = 6; **P < 0.01, ***P < 0.001 compared with 4-month-old mouse brain). (d) Quantification of immunoreactivity in (b) (mean ± SEM; one-way ANOVA, n = 6). (e) Western blot analysis of BIN1, BIN1 (1–277), and BIN1 (1–288) in tau P301S mouse brain. (f) Quantification of immunoreactivity in (e) (mean ± SEM; one-way ANOVA, n = 4; *P < 0.05, ***P < 0.001 compared with 2-month-old mouse brain). Source data can be found in S1 Data and S1 Raw Images. BIN1, bridging integrator 1; FL, full-length; WB, western blot. (TIF) [file pbio.3002470.s003.tif]

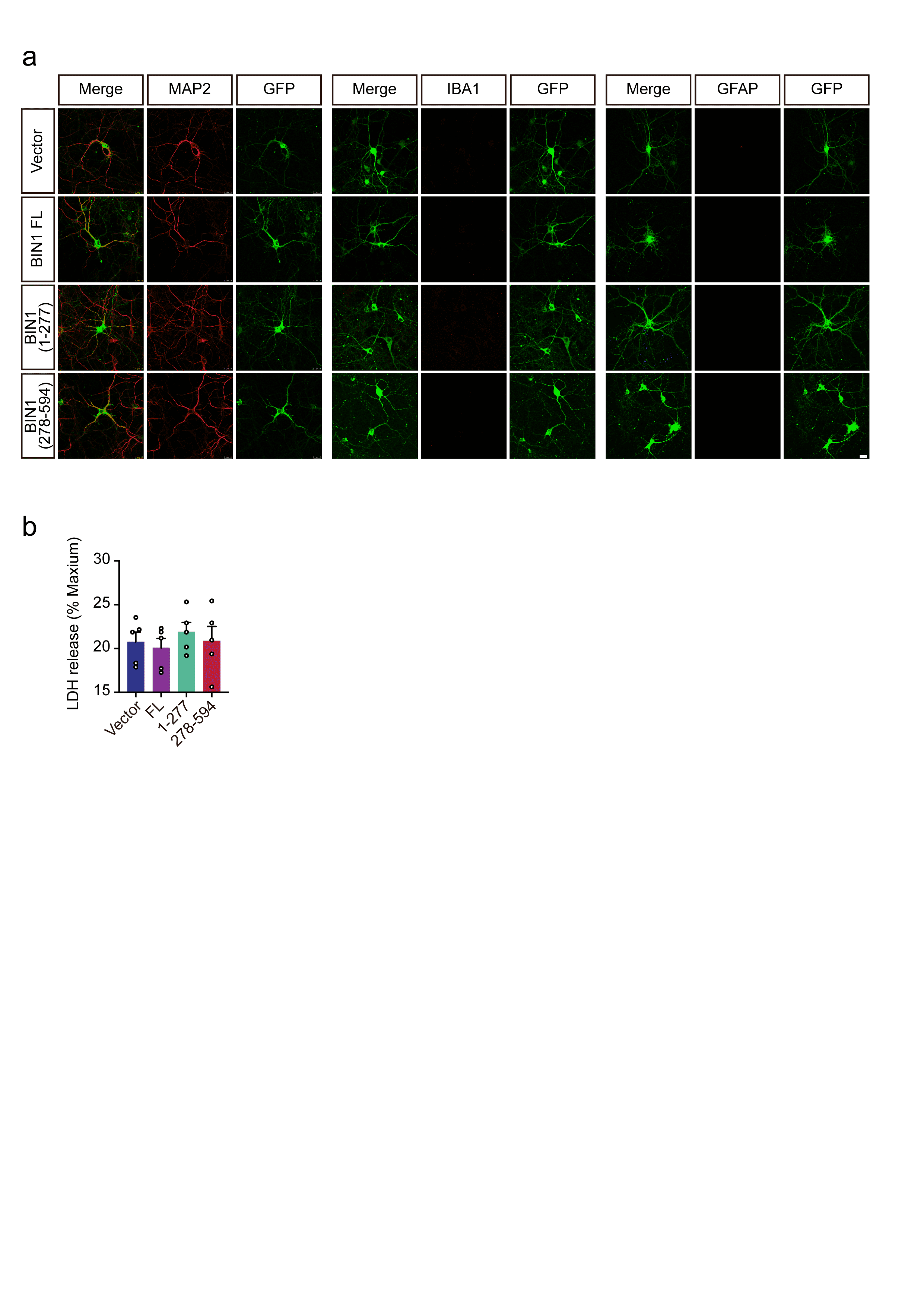

Supplement: S4 Fig — (a) Immunofluorescence staining of the neuronal marker MAP2, astrocyte marker GFAP, or microglial marker Iba1. Scale bar, 25 μm. (b) LDH release assay of neurons expressing full-length and fragmented BIN1 (mean ± SEM; one-way ANOVA, n = 5 independent experiments). Source data can be found in S1 Data. BIN1, bridging integrator 1; FL, full-length; GFP, green fluorescent protein; LDH, Lactate Dehydrogenase. (TIF) [file pbio.3002470.s004.tif]

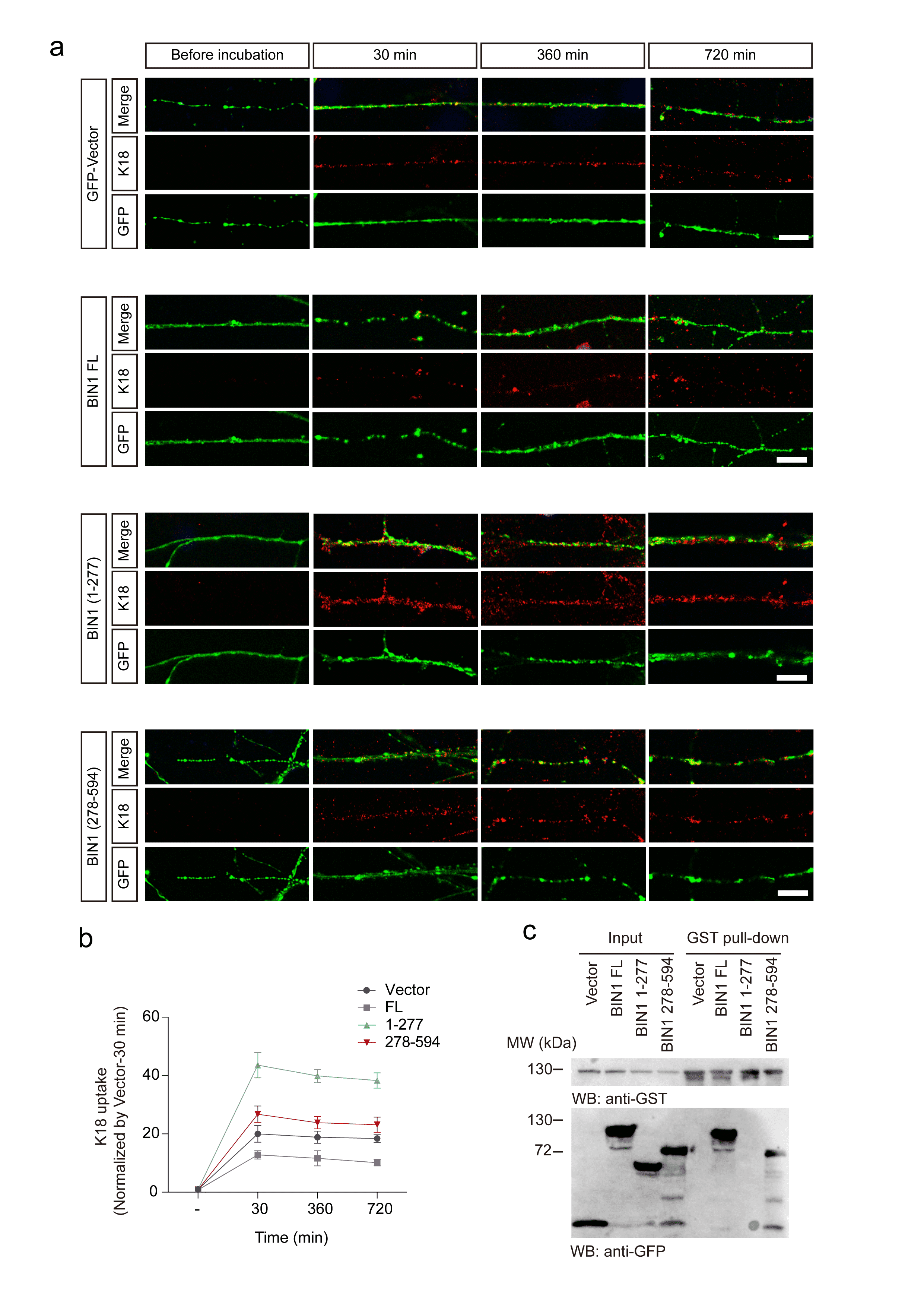

Supplement: S5 Fig — (a) Neurons expressing BIN1, BIN1 (1–277), or BIN1 (278–594) were exposed to K18 fibrils and washed with PBS. The representative immunofluorescence images show the K18 signals in neurons at different time points (0, 30 min, 360 min, and 720 min) after washing. Scale bar, 10 μm. (b) Quantification of K18 signals in (a) (mean ± SEM n = 60 cells per group; ***P < 0.001). (c) His pull-down assay shows that dynamin interacts with BIN1 and BIN1 (278–594), but not BIN1 (1–277). Source data can be found in S1 Data and S1 Raw Images. BIN1, bridging integrator 1; FL, full-length; GFP, green fluorescent protein; GST, Glutathione S-Transferase; MW, molecular weight; WB, western blot. (TIF) [file pbio.3002470.s005.tif]

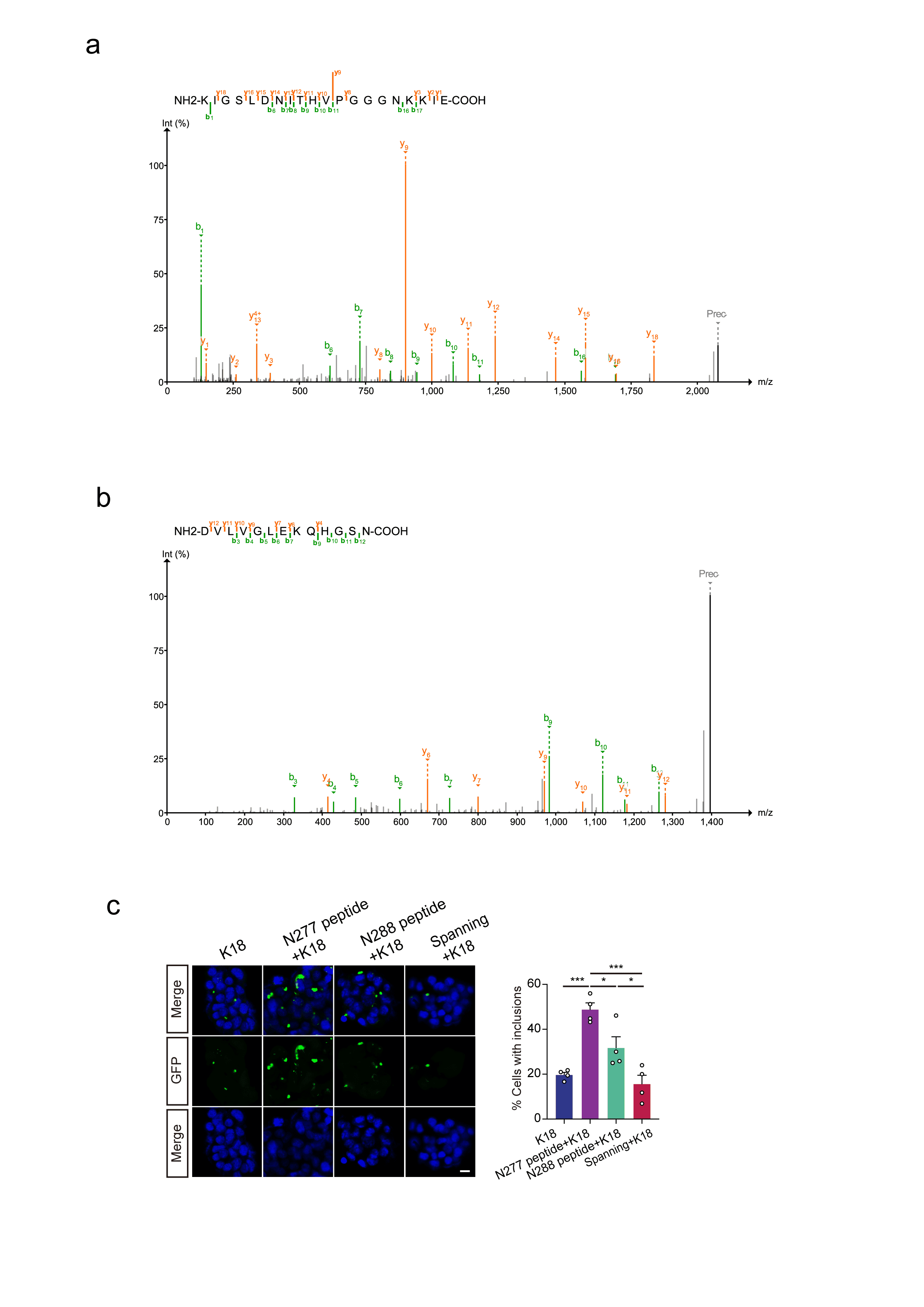

Supplement: S6 Fig — (a, b) MS/MS spectrum showing the identification of tau (a) and BIN1 N277 peptide (b). (c) HEK293 cells stably expressing tau RD-GFP were transduced with fibrils formed by K18 in the presence or absence of BIN1 N277 peptide, BIN1 N288 peptide, and BIN1 spanning peptide and permeabilized with 1% Triton X-100. The green dots indicate insoluble tau aggregates. Scale bar, 20 μm. (h) Quantification of the percentage of cells containing aggregates in cells. Data represent mean ± SEM of 4 independent experiments. *P < 0.05, ***P < 0.001. BIN1, bridging integrator 1; GFP, green fluorescent protein; RD, repeat domain. (TIF) [file pbio.3002470.s006.tif]

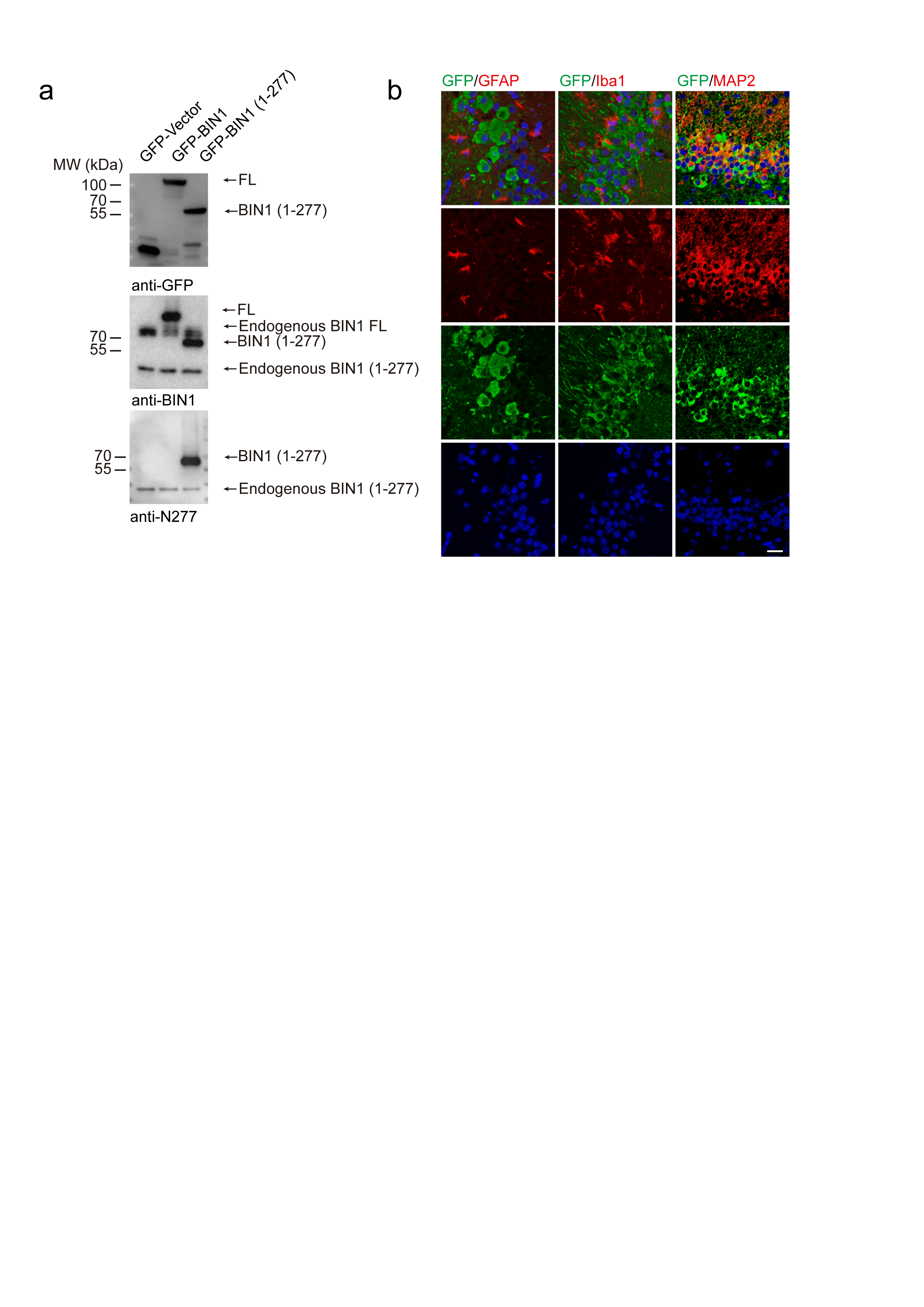

Supplement: S7 Fig — (a) Western blot showing the levels of BIN1 and BIN1 (1–277) in tau P301S mice. (b) Immunofluorescence showing that GFP-BIN1 fragments are mainly expressed in neurons, but not in astrocytes and microglia. Scale bar, 20 μm. Source data can be found in S1 Raw Images. BIN1, bridging integrator 1; FL, full-length; GFP, green fluorescent protein; MW, molecular weight. (TIF) [file pbio.3002470.s007.tif]

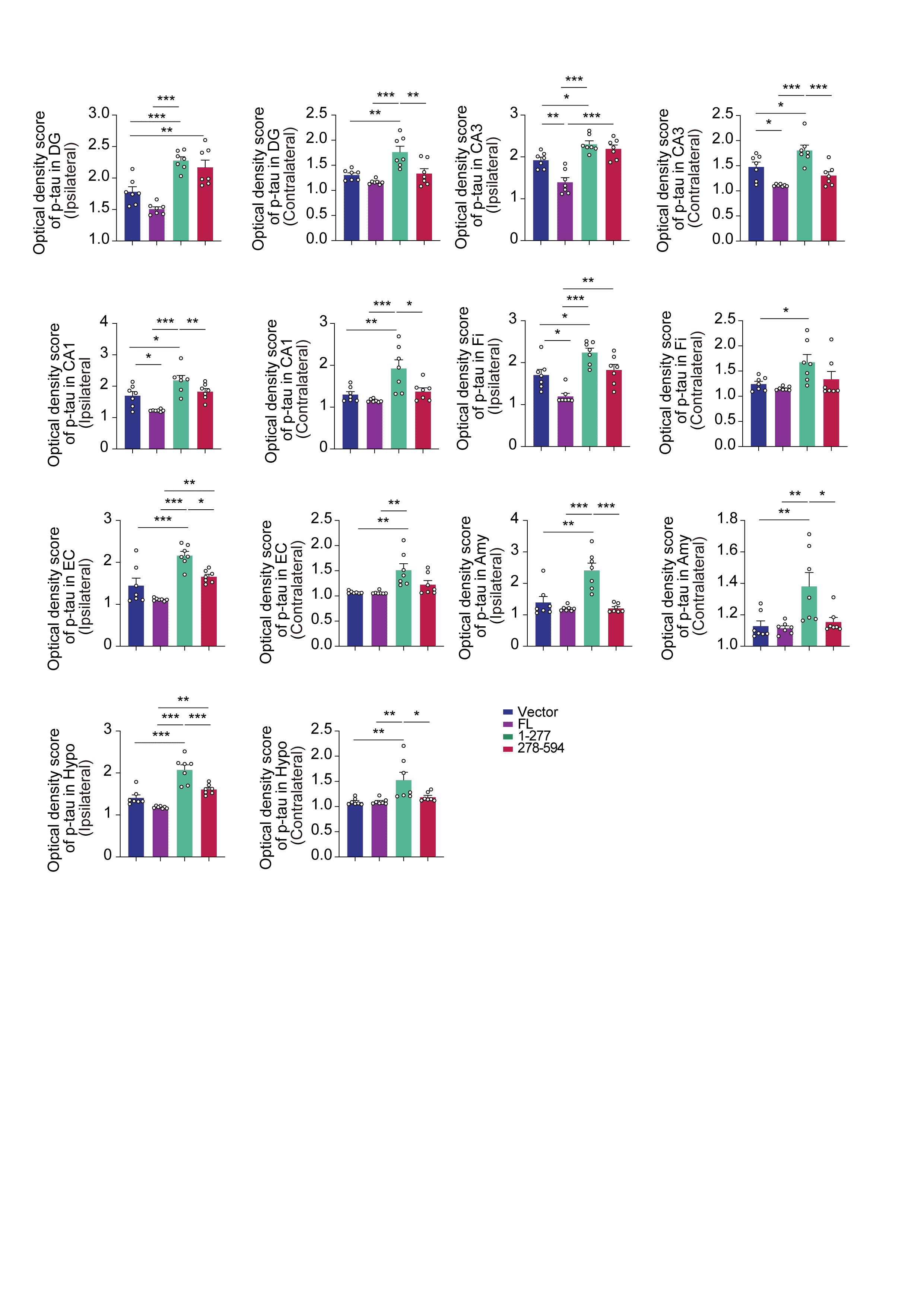

Supplement: S8 Fig — Mean ± SEM; n = 7 mice per group, *P < 0.05, **P < 0.01. ***P < 0.001. Source data can be found in S1 Data. AAV, adeno-associated virus; BIN1, bridging integrator 1; DG, dentate gyrus; EC, entorhinal cortex; FL, full-length; Fi, fimbria; p-tau, phospho-tau. (TIF) [file pbio.3002470.s008.tif]

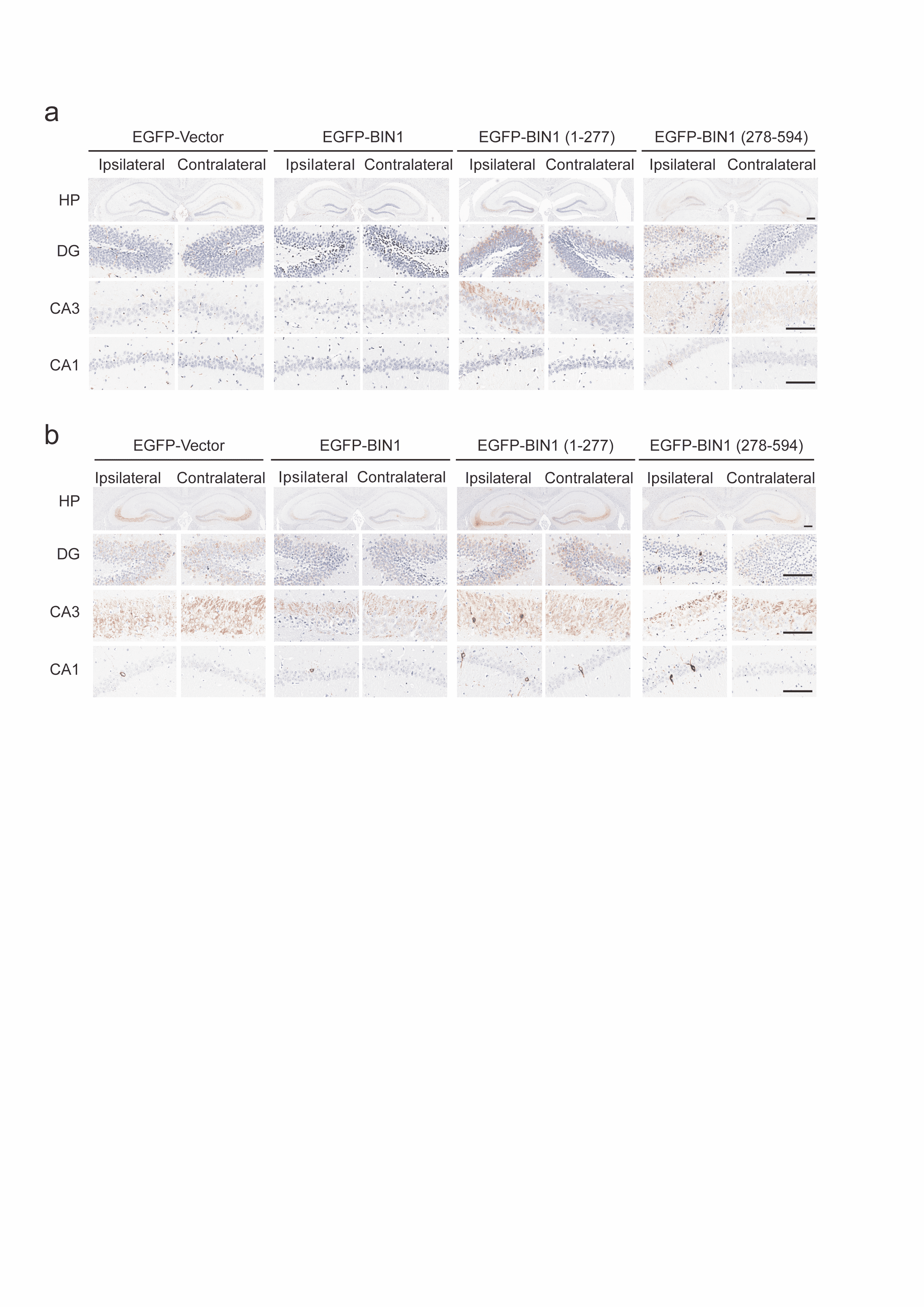

Supplement: S9 Fig — (a, b) AT8 immunostaining of the DG, CA3, and CA1 areas of the HP in tau P301S mice at 1 month (a) and 2 months (b) after injection of a mixture of K18 fibrils and AAVs encoding BIN1 FL, (1–277), or (278–594). Scale bar of the whole HP, 280 μm; Scale bar of DG, CA3, and CA1, 80 μm. AAV, adeno-associated virus; BIN1, bridging integrator 1; DG, dentate gyrus; EGFP, enhanced green fluorescent protein; FL, full-length; HP, hippocampus. (TIF) [file pbio.3002470.s009.tif]

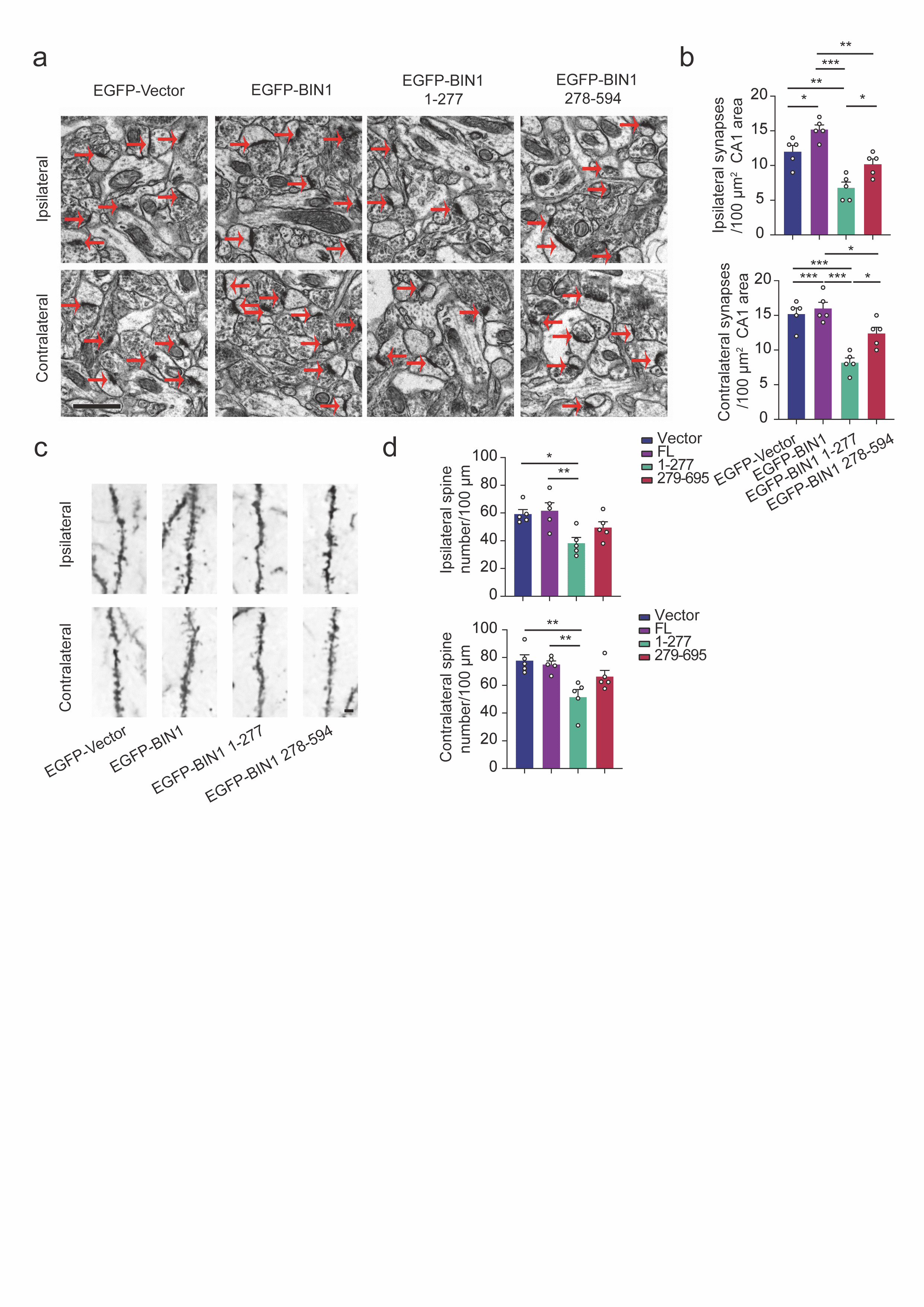

Supplement: S10 Fig — (a) Electron microscopy of synapses. Arrows indicate synapses. Scale bar, 2 μm. (b) Quantification of synaptic density (mean ± SEM; n = 5 mice per group; *P < 0.05, ***P < 0.001, one-way ANOVA). (c) Golgi staining revealed the dendritic spines from the apical dendritic layer of the CA1 region. Scale bar, 2.5 μm. (d) Quantification of spine density (mean ± SEM; n = 5 mice per group; **P < 0.01, one-way ANOVA). Source data can be found in S1 Data. BIN1, bridging integrator 1; EGFP, xxxx; FL, xxxx. (TIF) [file pbio.3002470.s010.tif]

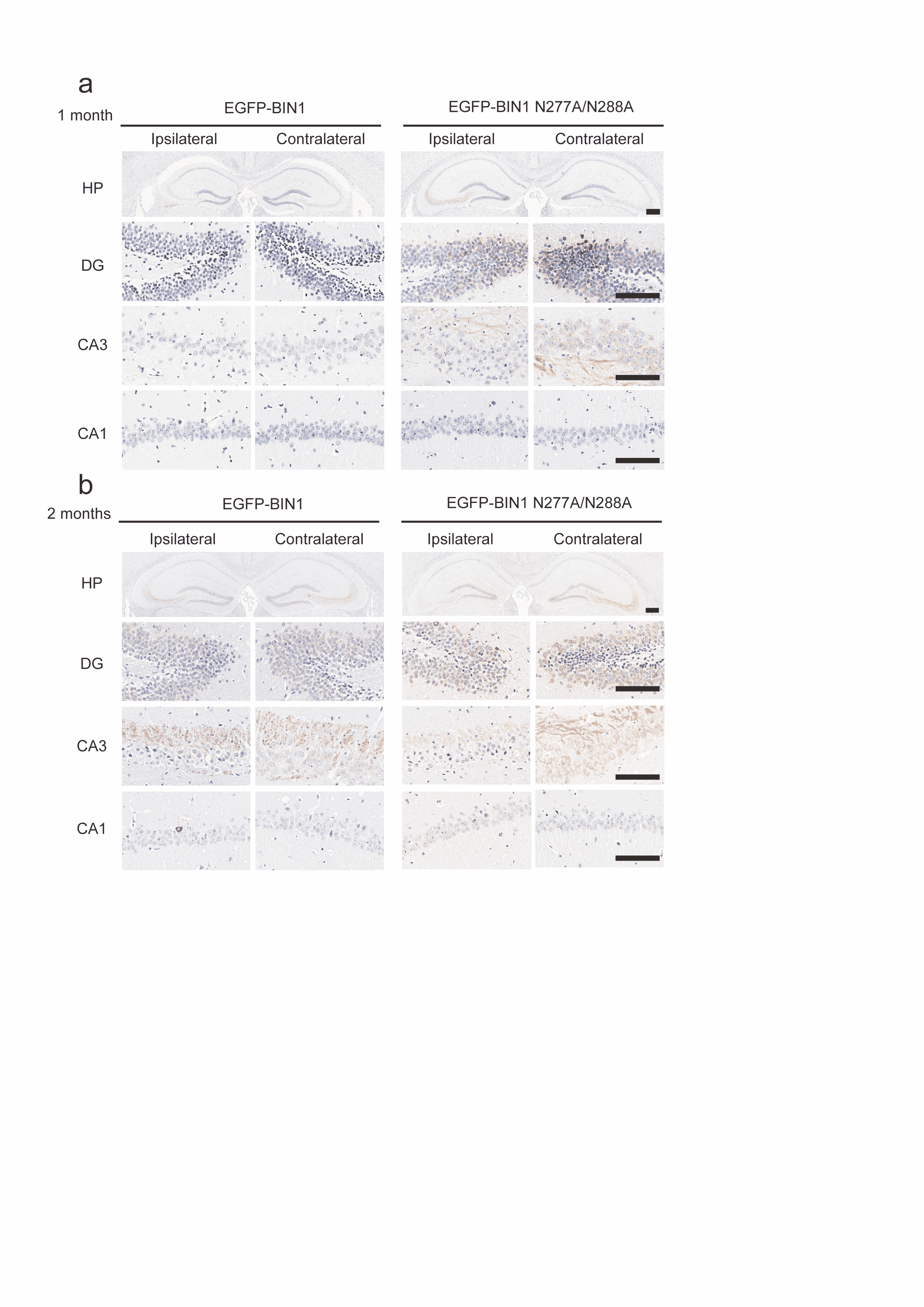

Supplement: S11 Fig — (a, b) AT8 immunostaining of DG, CA3, and CA1 areas of the HP in tau P301S mice at 1 month (a) and 2 months (b) after injection of a mix of K18 fibrils and AAVs encoding wild-type or N277A/N288A mutant BIN1. Scale bar of the whole HP, 280 μm; Scale bar of DG, CA3, and CA1, 140 μm. AAV, adeno-associated virus; BIN1, bridging integrator 1; DG, dentate gyrus; EGFP, xxxx; HP, hippocampus. (TIF) [file pbio.3002470.s011.tif]

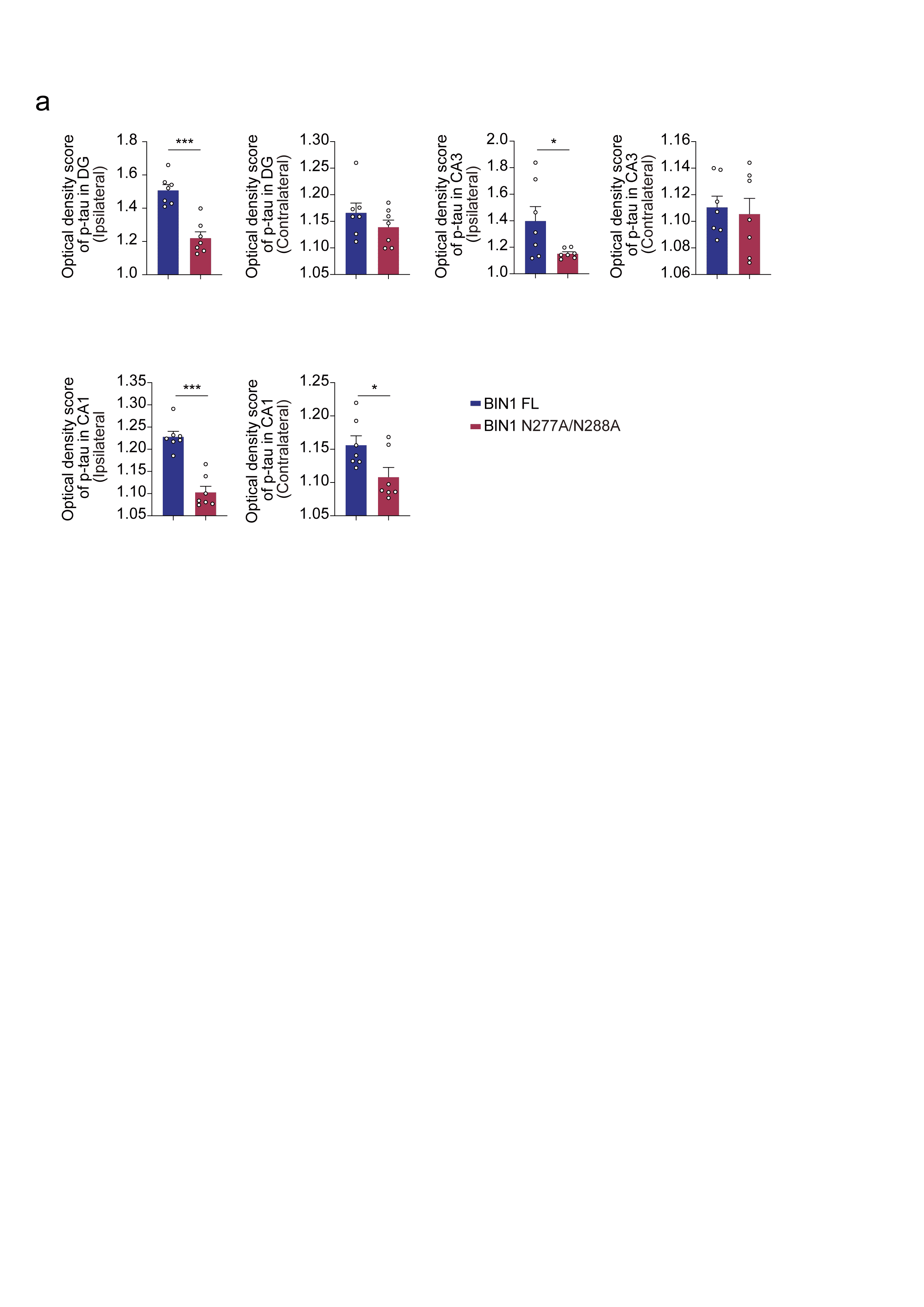

Supplement: S12 Fig — Mean ± SEM; n = 7 mice per group, *P < 0.05, **P < 0.01. ***P < 0.001. Source data can be found in S1 Data. AAV, adeno-associated virus; BIN1, bridging integrator 1; DG, dentate gyrus; FL, full-length; p-tau, phospho-tau. (TIF) [file pbio.3002470.s012.tif]

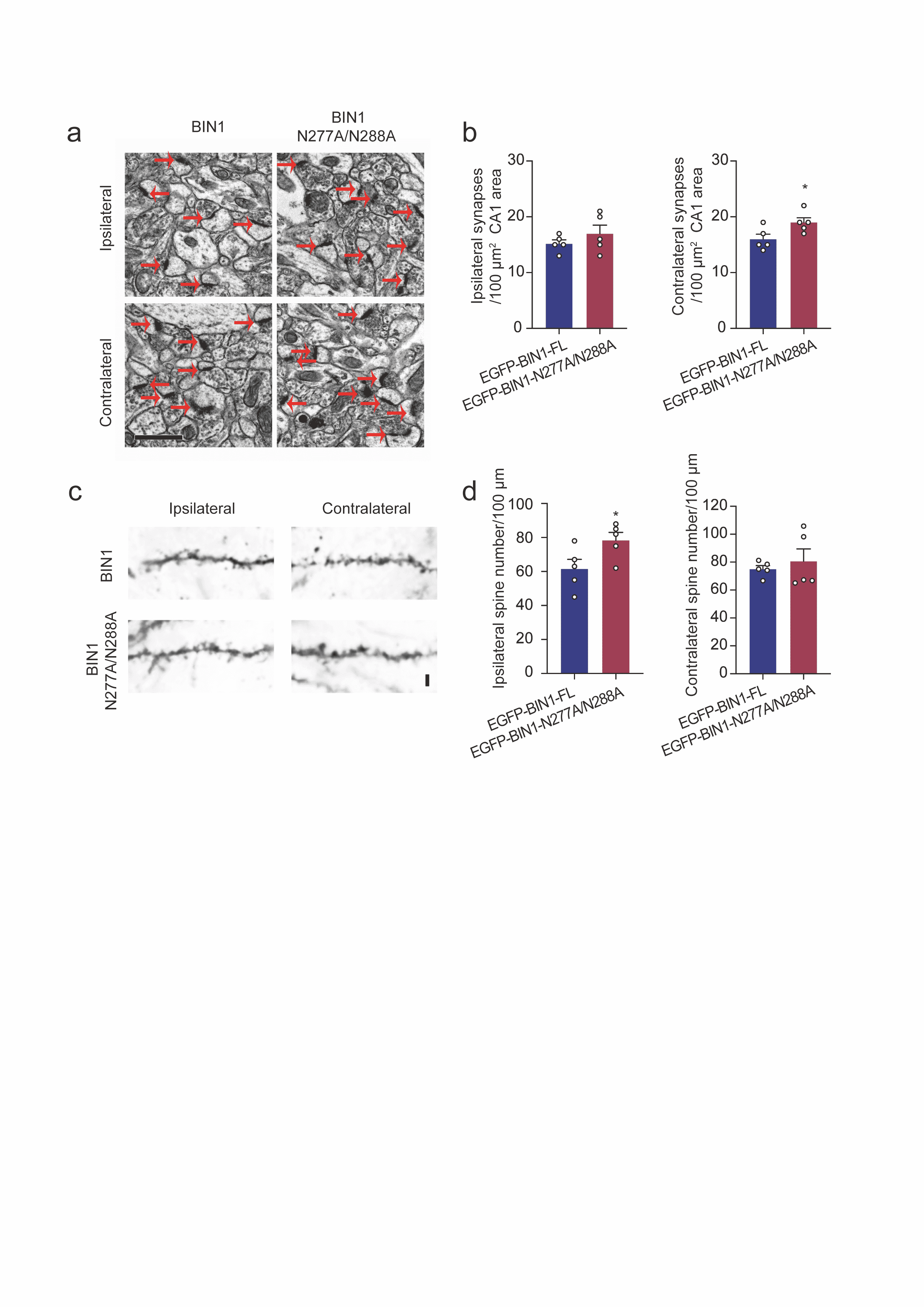

Supplement: S13 Fig — (a) Electron microscopy of synapses. Arrows indicate synapses. Scale bar, 2 μm. (b) Quantification of synaptic density (mean ± SEM; n = 5 mice per group; *P < 0.05, Student t test). (c) Golgi staining revealed the dendritic spines from the apical dendritic layer of the CA1 region. Scale bar, 2.5 μm. (d) Quantification of spine density (mean ± SEM; n = 5; *P < 0.05, Student t test). Source data can be found in S1 Data. BIN1, bridging integrator 1; EGFP, enhanced green fluorescent protein; FL, full-length. (TIF) [file pbio.3002470.s013.tif]

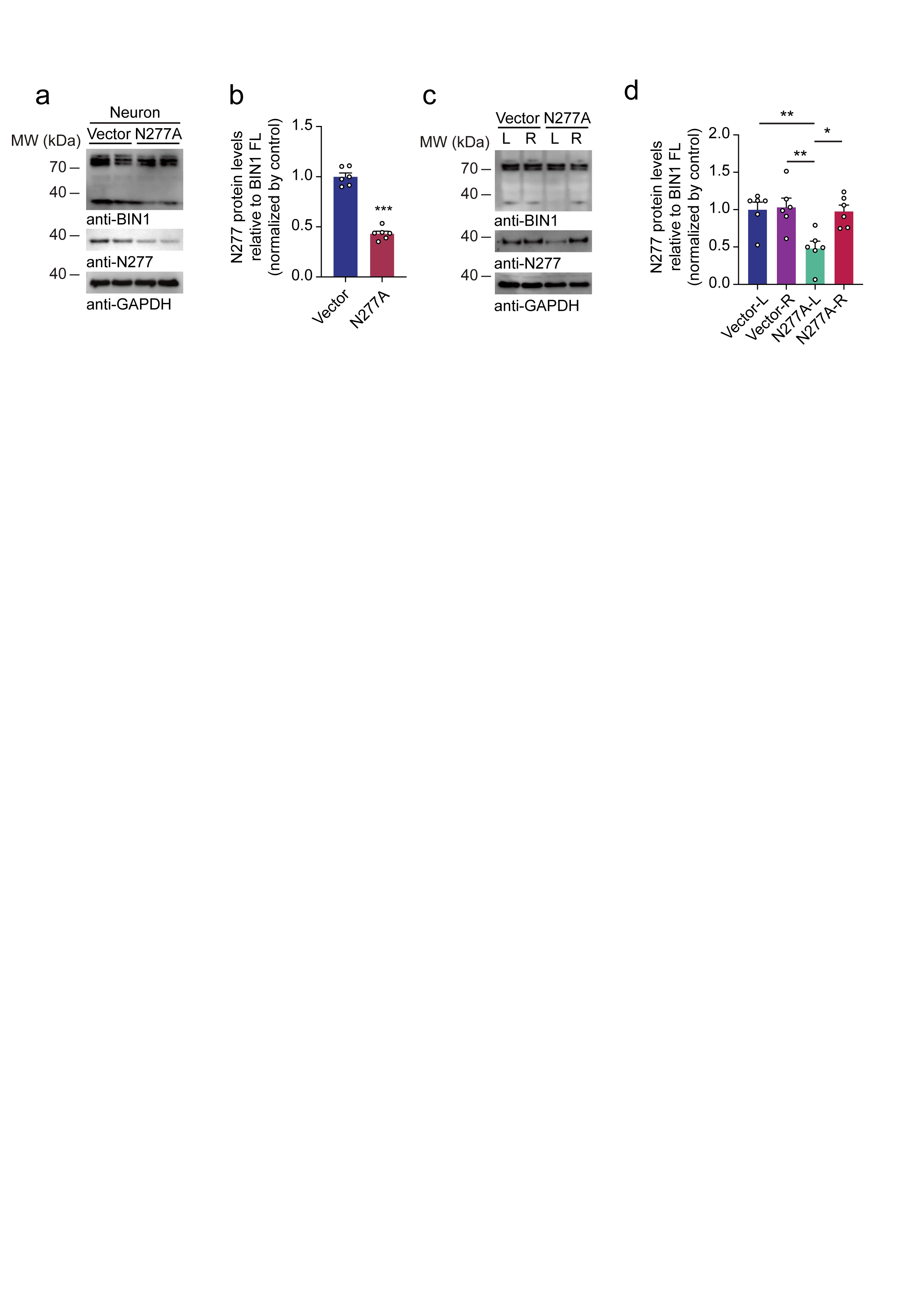

Supplement: S14 Fig — (a, b) The expression of BIN1 (1–277) in primary neurons infected with control AAVs (vector) or AAVs mediating the mutation (N277A). (c, d) The control AAVs (vector) or AAVs mediating the mutation (N277A) were injected into the left hippocampus of tau P301S mice. Western blot shows the expression of BIN1 (1–277) in brain lysates (mean ± SEM; one-way ANOVA, n = 6). *P < 0.05, **P < 0.01, ***P < 0.001. L, left; R, right. Source data can be found in S1 Data and S1 Raw Images. AAV, adeno-associated virus; BIN1, bridging integrator 1; FL, full-length; MW, molecular weight. (TIF) [file pbio.3002470.s014.tif]

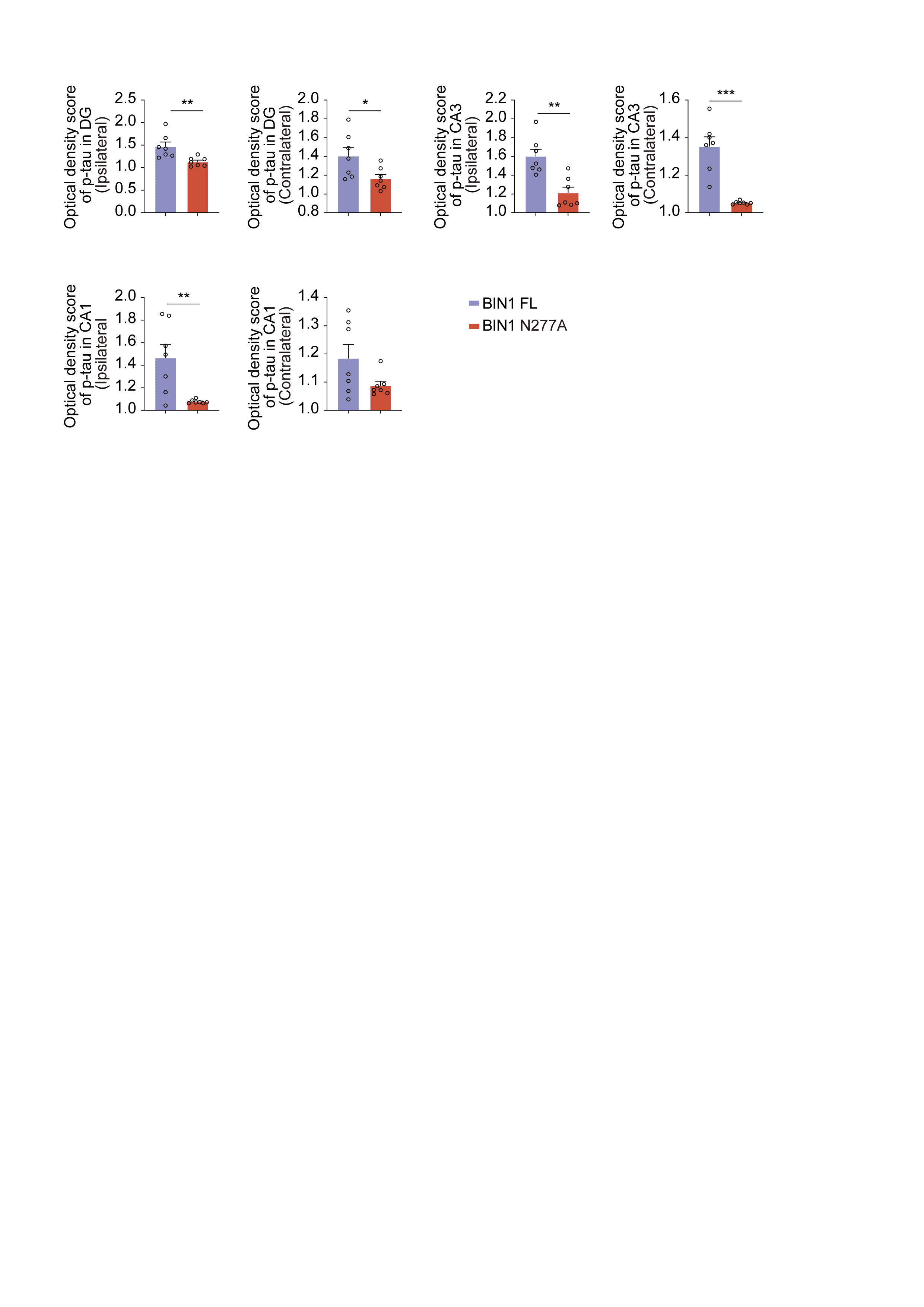

Supplement: S15 Fig — Mean ± SEM; n = 7 mice per group, *P < 0.05, **P < 0.01. ***P < 0.001. Source data can be found in S1 Data. AAV, adeno-associated virus; BIN1, bridging integrator 1; DG, dentate gyrus; FL, xxxx; p-tau, phospho-tau. (TIF) [file pbio.3002470.s015.tif]

Figure 1

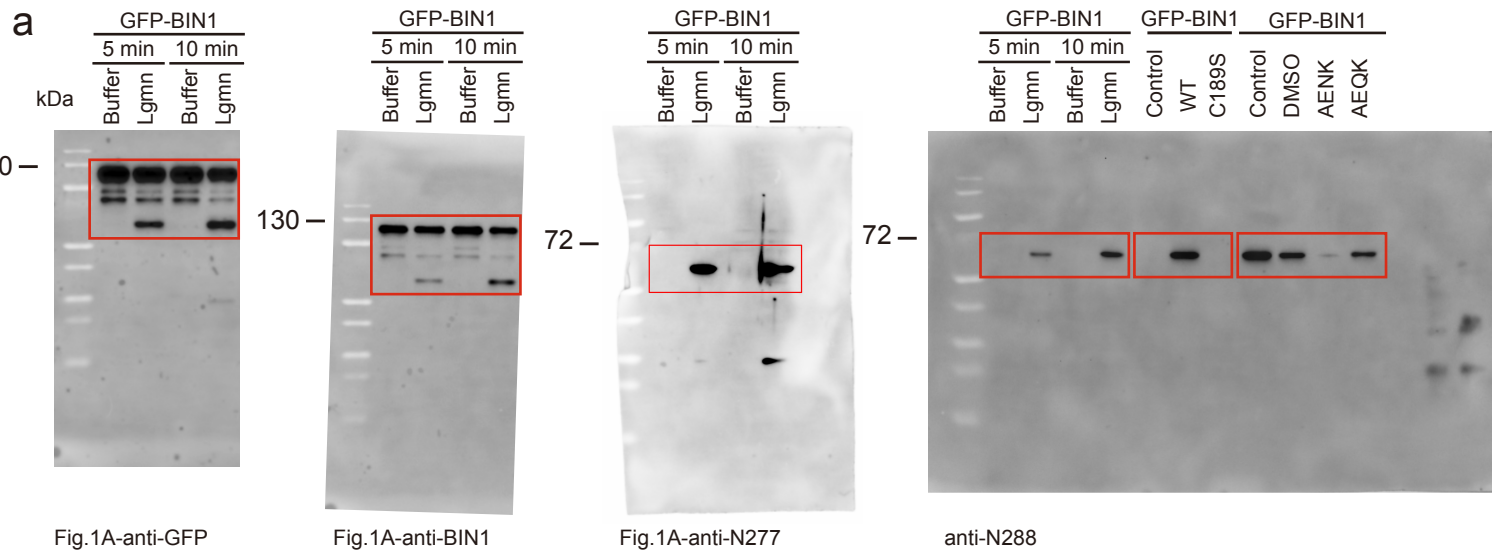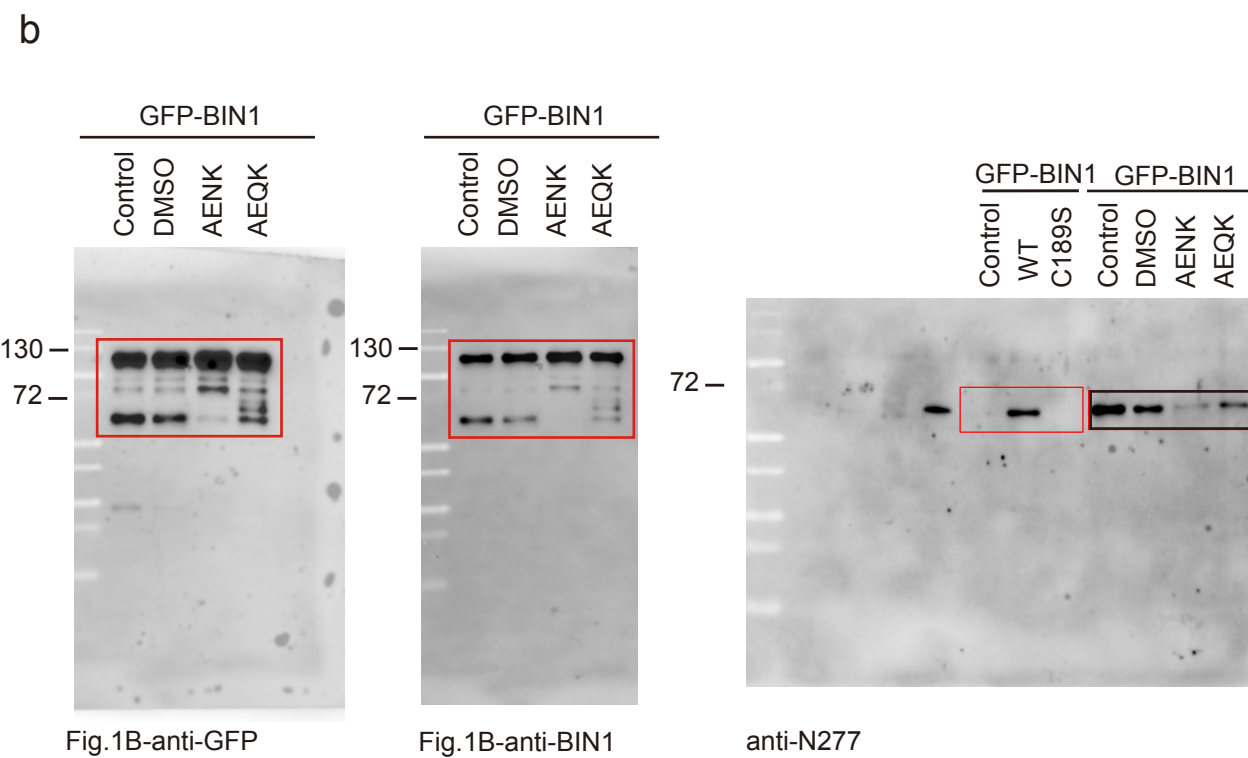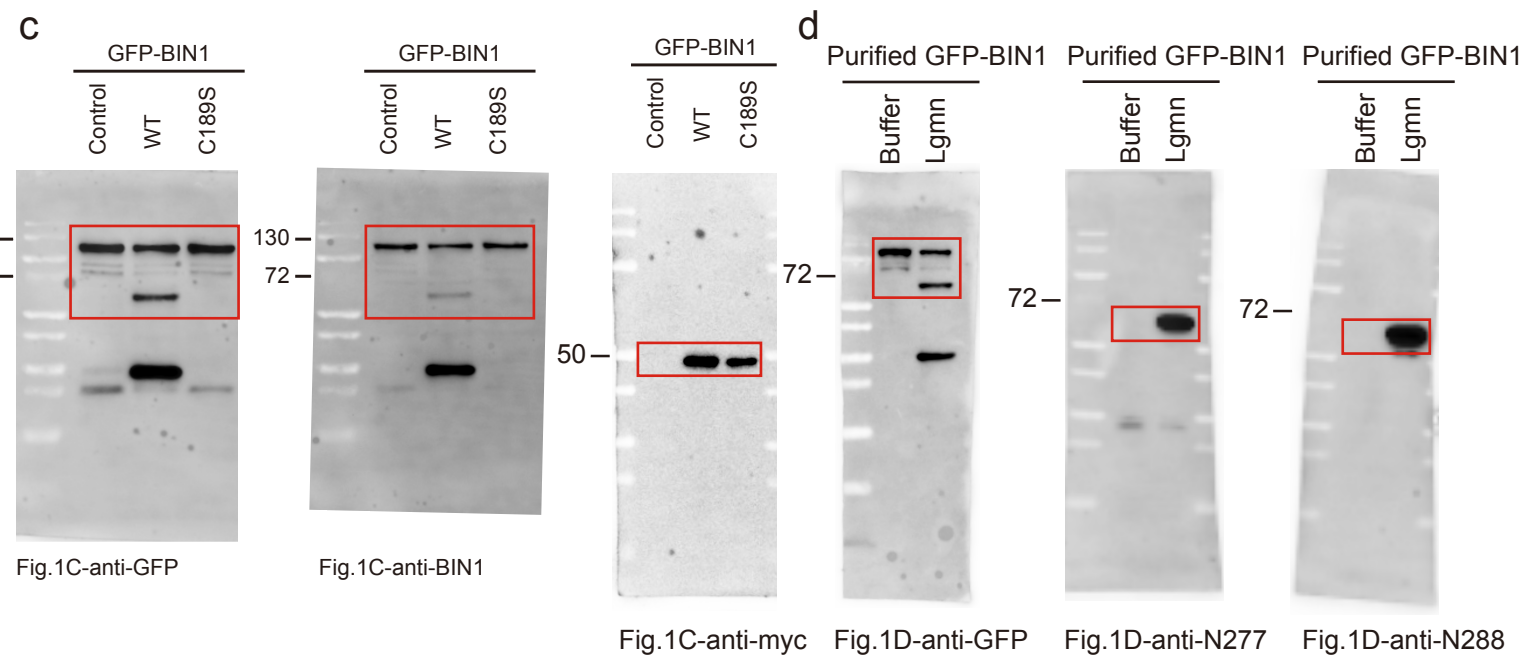

Figure 2

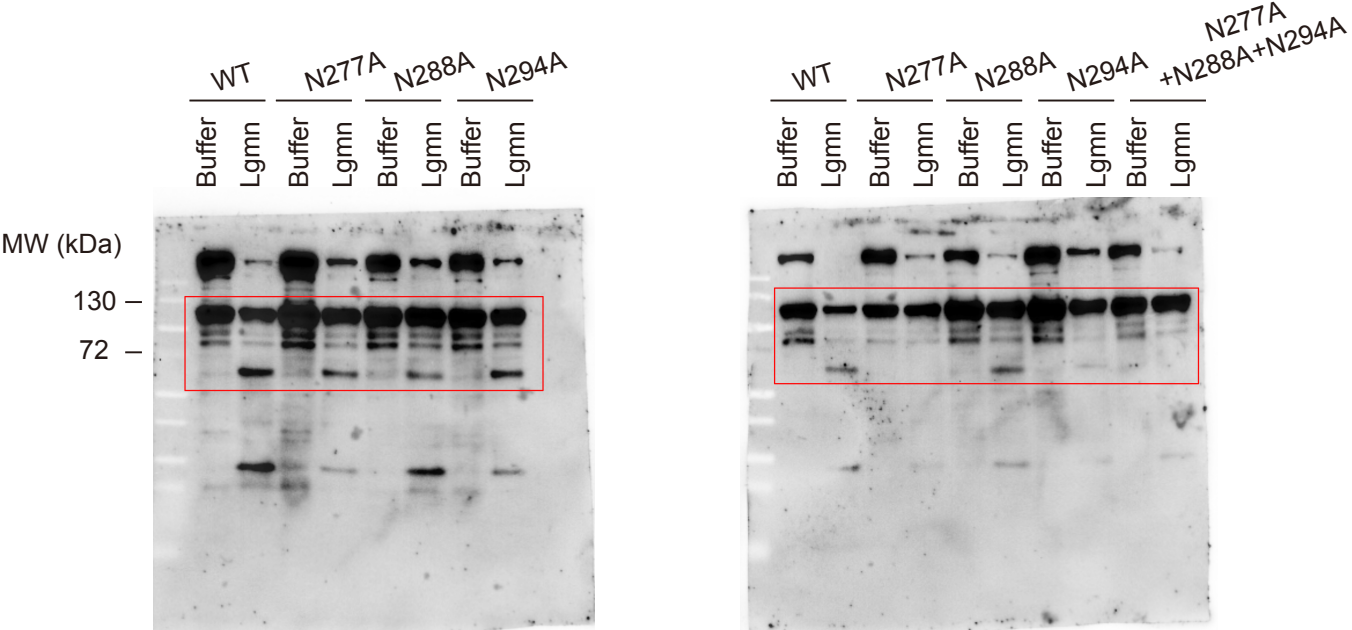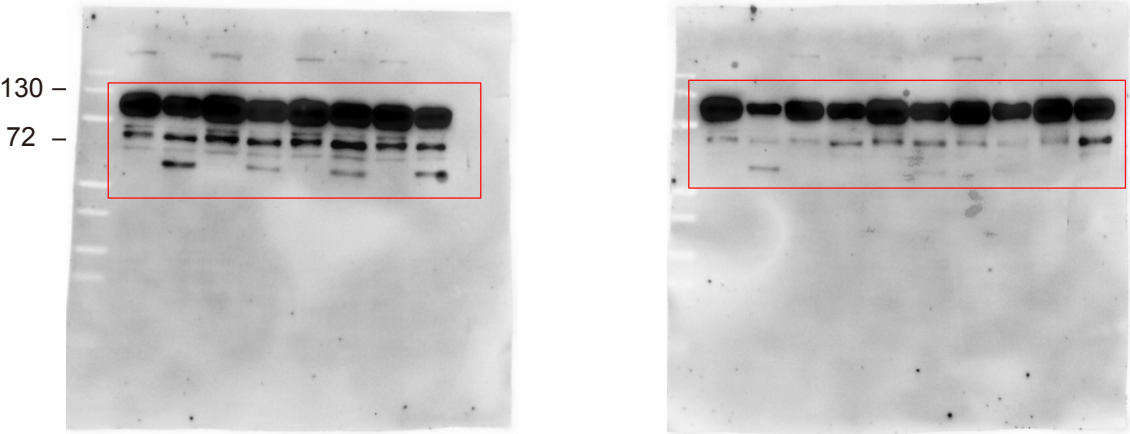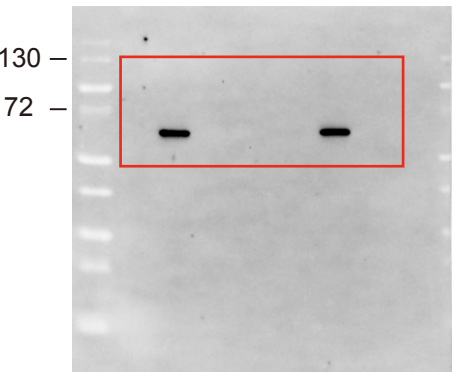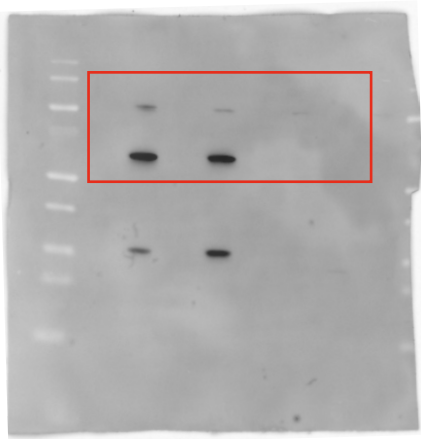

Figure 5

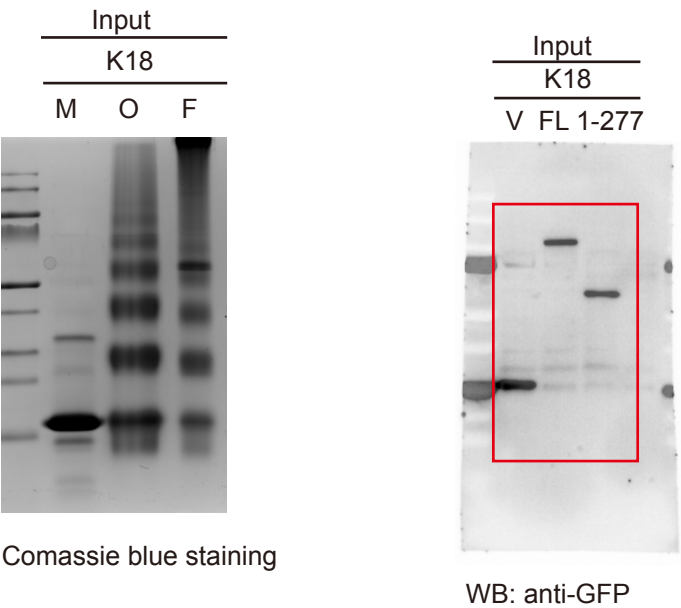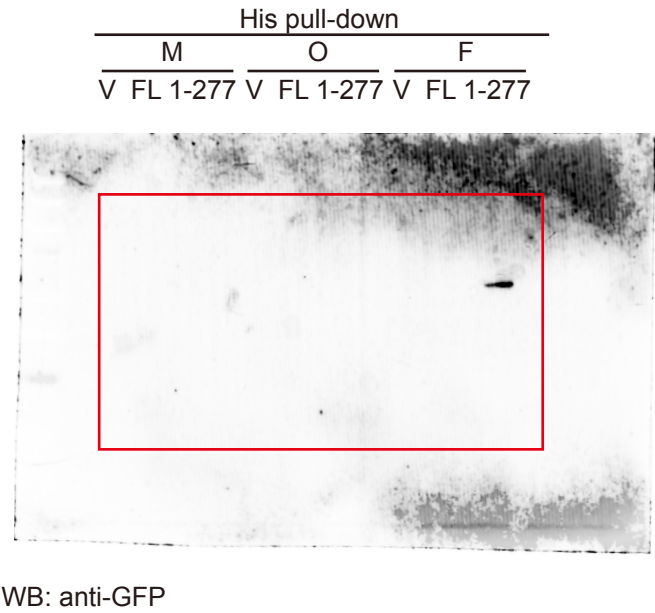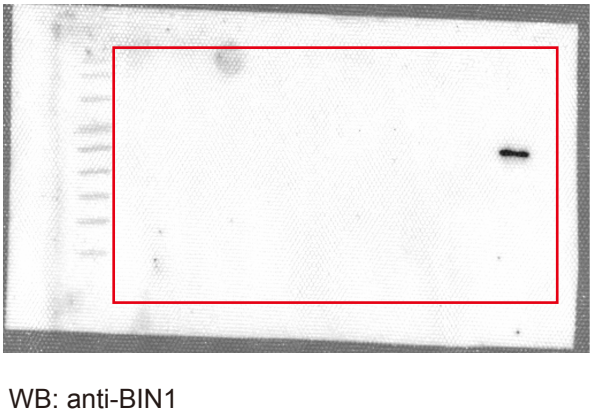

Figure S1

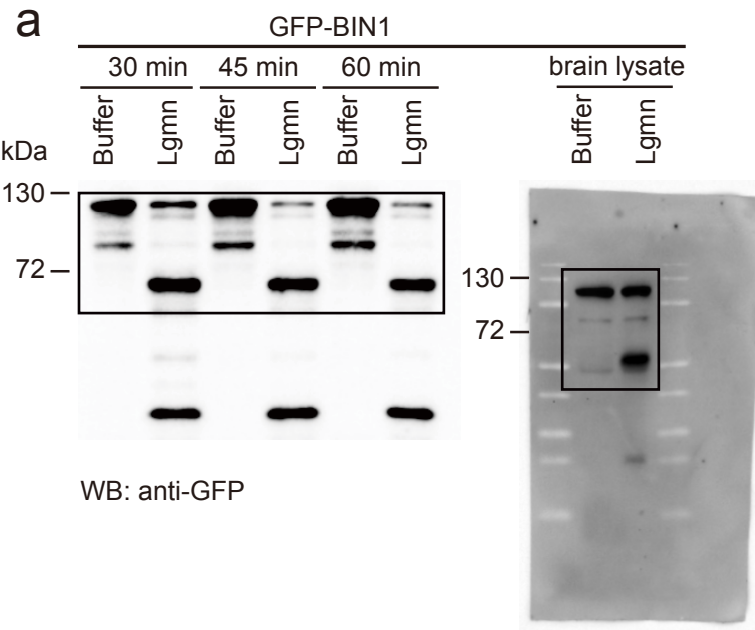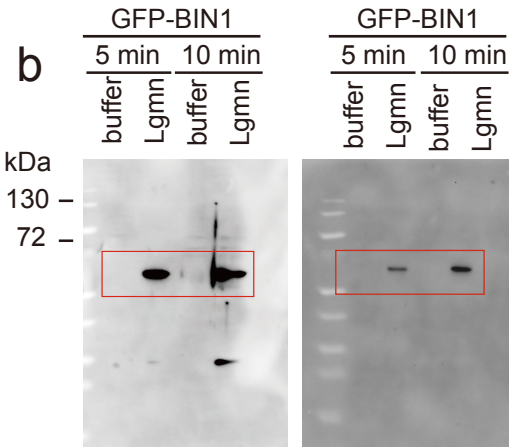

Figure S2

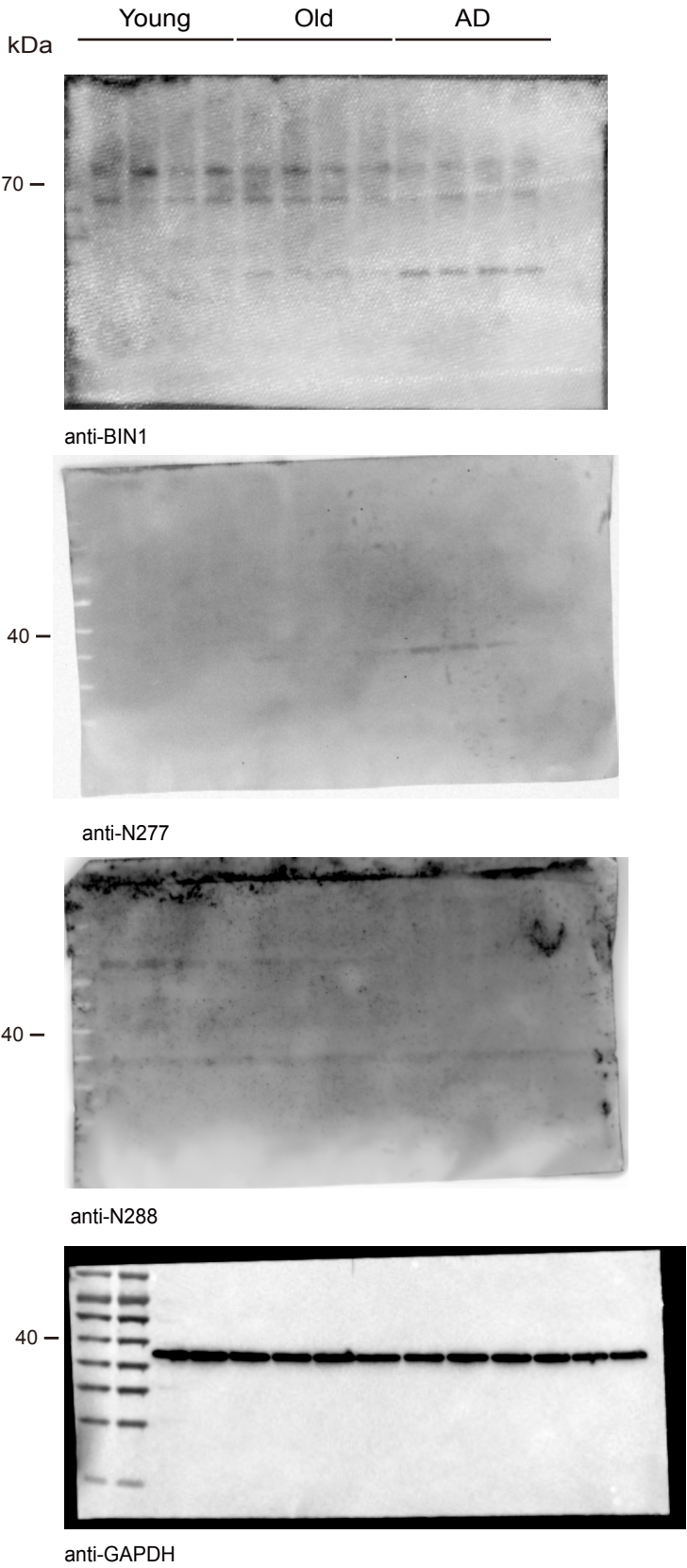

Figure S3

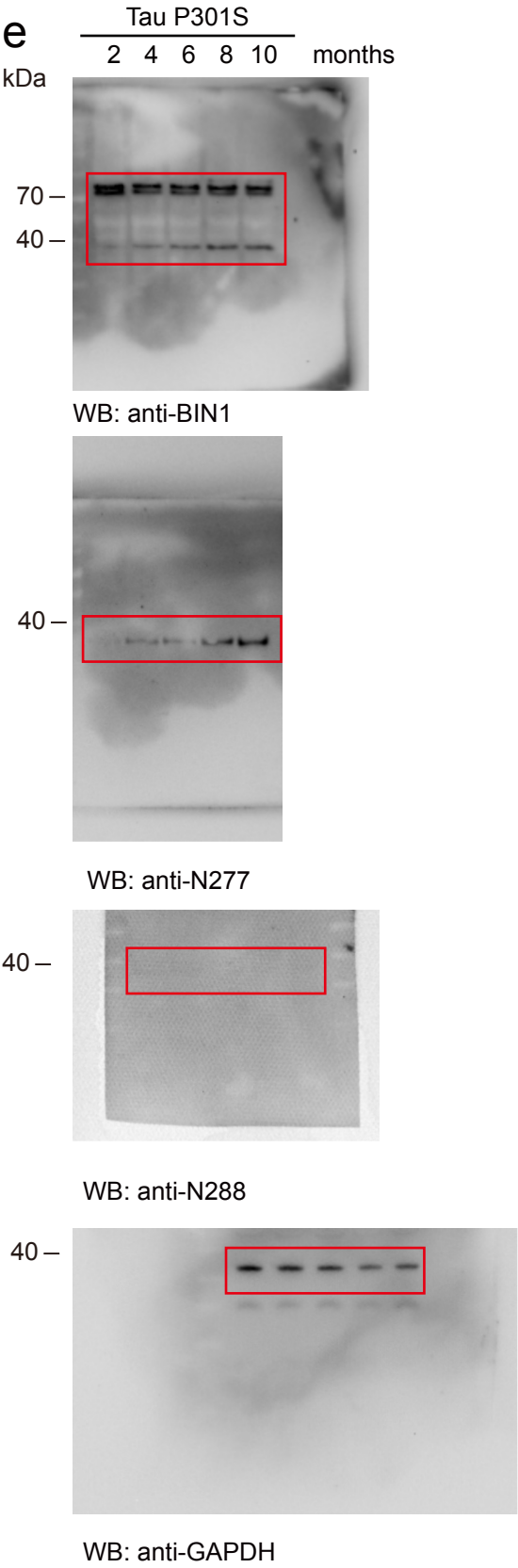

### Figure S5

C

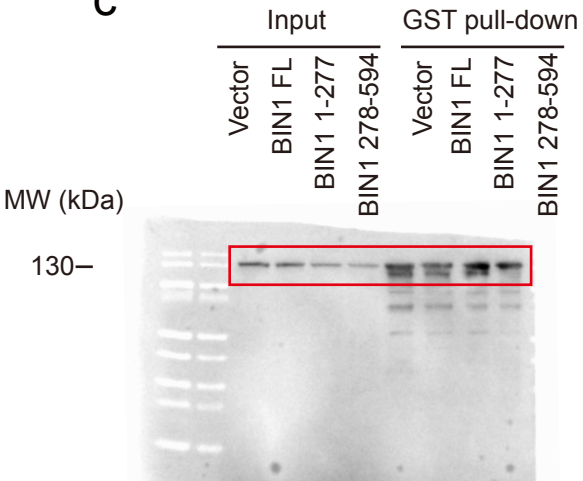

WB: anti-GST

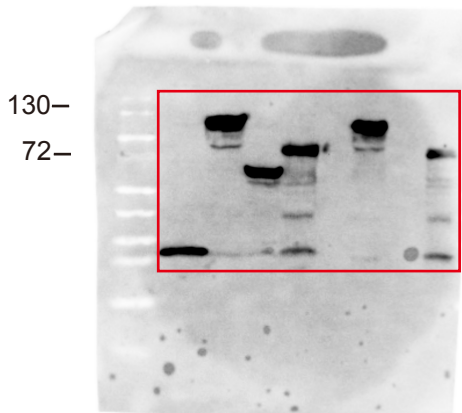

WB: anti-GFP

Figure S7

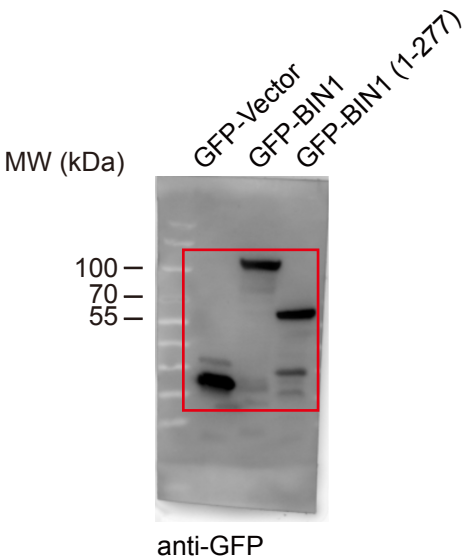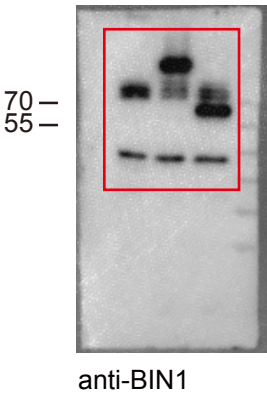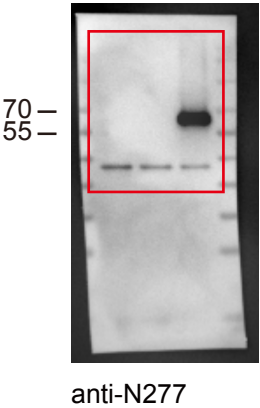

Figure S14

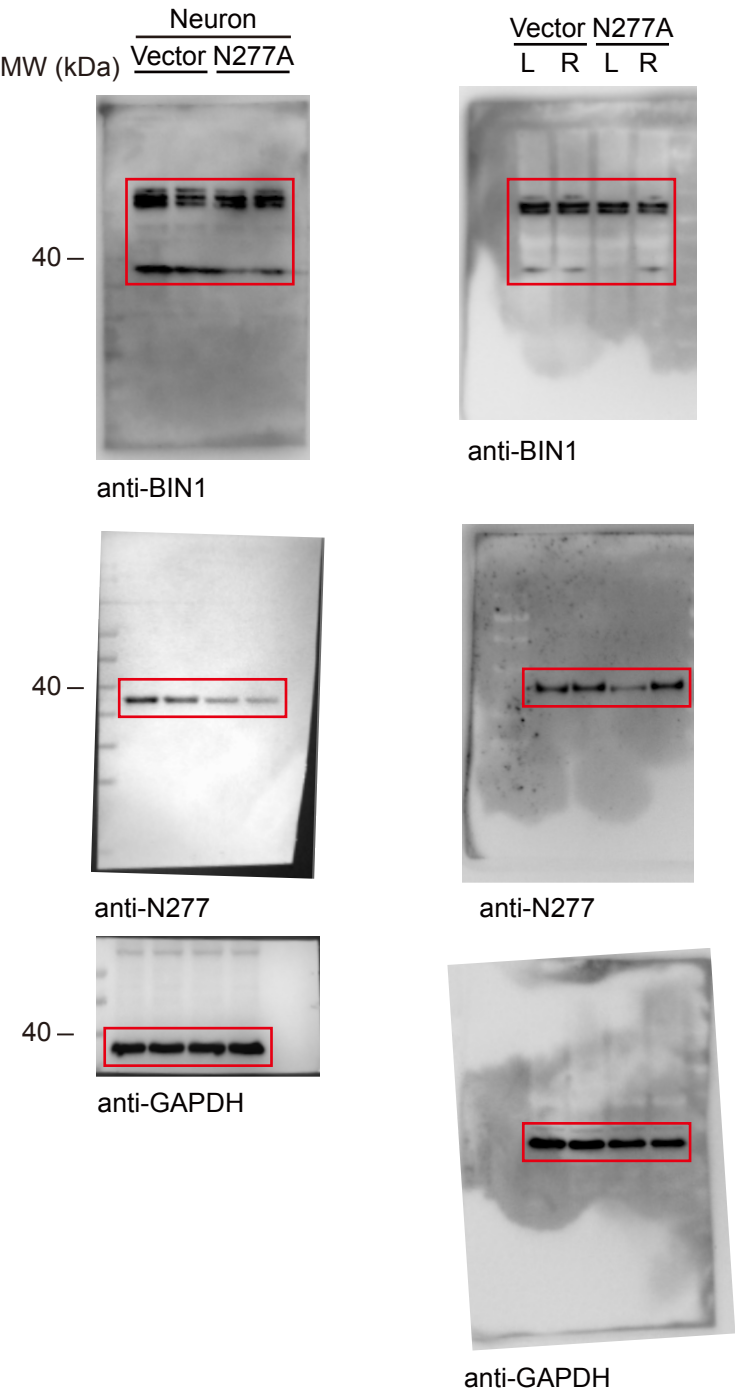

Supplement: S1 Raw images — (PDF) [file pbio.3002470.s018.pdf]
