# Supplementary material for: Bridging integrator 1 fragment accelerates tau aggregation and propagation by enhancing clathrin-mediated endocytosis in mice
Source: PLoS Biol. 2024 Jan 11;22(1):e3002470. doi: 10.1371/journal.pbio.3002470 (PMC10783739; doi:10.1371/journal.pbio.3002470)
Supplement: S1 Table — (DOCX) [file pbio.3002470.s016.docx]

**Supplementary table 1. The neuropathological characteristics of human samples.**

| **Primary Neuropathologic Diagnosis** | **Braak Stage** | **PMI (hr)** | **Age at Death** | **Disease Duration (years)** | **Race**  **/sex** | **ApoE** |
| --- | --- | --- | --- | --- | --- | --- |
| **Control** | 0 | 3 | 52 | N/A | wf | E3/4 |
| **Control** | 0 | 6 | 59 | N/A | bm | E2/3 |
| **Control** | 0 | 6 | 65 | N/A | wf | E3/3 |
| **Control** | 0 | N/A | 70 | N/A | wm | E2/3 |
| **Control** | I | 2.5 | 70 | N/A | bm | E3/3 |
| **Control** | 0 | N/A | 61 | N/A | bm | E3/4 |
| **Control** | II | 5.5 | 94 | N/A | wm | E3/3 |
| **Control** | II | <12 | 61 | N/A | bm | E3/4 |
| **Control** | II | 7 | 74 | N/A | wf | E3/3 |
| **Control** | III | 6 | 91 | N/A | wf | E3/3 |
| **Control** | III | 15.5 | 92 | N/A | wf | E3/3 |
| **AD** | III | 17.5 | 70 | 8 | wm | E3/3 |
| **AD** | VI | 5.5 | 54 | 9 | wm | E3/3 |
| **AD** | VI | 6 | 62 | 10 | wf | E3/3 |
| **AD** | VI | 6 | 62 | 7 | wm | E2/3 |
| **AD** | V | 4.5 | w | 3 | wf | E3/4 |
| **AD** | V | N/A | 77 | 7 | wf | E3/4 |
| **AD** | V | 11.5 | 62 | 10 | wm | E3/4 |
