# Supplementary material for: Bridging integrator 1 fragment accelerates tau aggregation and propagation by enhancing clathrin-mediated endocytosis in mice
Source: PLoS Biol. 2024 Jan 11;22(1):e3002470. doi: 10.1371/journal.pbio.3002470 (PMC10783739; doi:10.1371/journal.pbio.3002470)
Supplement: S2 Table — (DOCX) [file pbio.3002470.s017.docx]

**Supplementary table 2. The sequence of sgRNA, the donor sequence, and the primer sequences for PCR amplification of genomic DNA (gDNA)**

| Name | Sequence (5’-3’) |
| --- | --- |
| sgRNA | tgctcccgtgctgcttctctagg |
| Donor | aagaaataaaatgtcgacatgccttatgctttaaatccagcaagtaggaagactaaggtaaagaattgtgagtctgaggccagggcagctttctgagagattatctcaaactaaaataaacaaaaagagctggggctgtgggccagtagtagagtacttgcctaacatgtaaagcctttggctcagccaaaggctttgaatatgttaggtattctacttccccctgccccaaacagtagactcttgtgtggcatttaggatatccctttaaatagcaagagagaaaagatgaccccagagtcagtagctgtgagcaatacggctcacatcccctggtttctatgccatcagttggctttctgggttcactgtgctggtatcatcttccgttggtggagtcaggggaagctgctcctgggagaggcaggggaaggagcgaggaactattcctgacgaagatgctcgtccaagcctgcttctcttcactcttcctcctgcagctcaatcagaacctcaatgatgtcctggtcagtctggagaagcagcacgggagcgctaccttcacagtcaaggcccaacccaggtaggttagggcagggagggtgaggtcagtggggccctgtggcatgatggtcagcgccacagaagcagcttctggagacccttccttgttcttctcgcgggcctctggaaatcttccagactctgcctcctggcatcagggaaatagctcctatagtcttggtctgtggccagggagcctgtagatcccagagccttagtagaatagaggtcaagatggagatggggctctattgttgacacaaggctgtggctcctggcatgcaggccttcctgcctcttgctcaaggtaaggtccaagaatggcagcagtgccttggtactttgtcctttgctgcctttcctgggcagctgccaggagctcctgtcacctgagggaggtcatgagggggaaggaatagaaacaacctggagtctgggattgcacgccttccccctccctccctctctgtctctcccttcttactccgtgtgtgtgtgtgtgtgtgtgtgtgtgtgtgt |
| Primer-FP | atgtcgacatgccttatgc |
| Primer-RP | cagactccaggttgtttcta |
